# Stat3 Signaling Promotes Survival And Maintenance Of Medullary Thymic Epithelial Cells

**DOI:** 10.1371/journal.pgen.1005777

**Published:** 2016-01-20

**Authors:** Dakshayani Lomada, Manju Jain, Michelle Bolner, Kaitlin A. G. Reeh, Rhea Kang, Madhava C. Reddy, John DiGiovanni, Ellen R. Richie

**Affiliations:** 1 Department of Epigenetics and Molecular Carcinogenesis, The University of Texas M.D. Anderson Cancer Center, Science Park, Smithville, Texas, United States of America; 2 Epigenetics and Molecular Carcinogenesis Graduate Program, The University of Texas Graduate School of Biomedical Sciences Houston, Texas, United States of America; 3 Division of Pharmacology and Toxicology, College of Pharmacy, The University of Texas at Austin, Austin, Texas, United States of America; University of Edinburgh, UNITED KINGDOM

## Abstract

Medullary thymic epithelial cells (mTECs) are essential for establishing central tolerance by expressing a diverse array of self-peptides that delete autoreactive thymocytes and/or divert thymocytes into the regulatory T cell lineage. Activation of the NFκB signaling pathway in mTEC precursors is indispensable for mTEC maturation and proliferation resulting in proper medullary region formation. Here we show that the Stat3-mediated signaling pathway also plays a key role in mTEC development and homeostasis. Expression of a constitutively active Stat3 transgene targeted to the mTEC compartment increases mTEC cellularity and bypasses the requirement for signals from positively selected thymocytes to drive medullary region formation. Conversely, conditional deletion of Stat3 disrupts medullary region architecture and reduces the number of mTECs. Stat3 signaling does not affect mTEC proliferation, but rather promotes survival of immature MHCII^lo^CD80^lo^ mTEC precursors. In contrast to striking alterations in the mTEC compartment, neither enforced expression nor deletion of Stat3 affects cTEC cellularity or organization. These results demonstrate that in addition to the NFkB pathway, Stat3-mediated signals play an essential role in regulating mTEC cellularity and medullary region homeostasis.

## Author Summary

T cells, an essential component of the immune system, generate protective immune responses against pathogenic organisms and cancer cells. T cells are produced in the thymus, which provides a unique microenvironment required for T cell development. Distinct subsets of thymic epithelial cells (TECs) in the outer cortex and inner medulla provide signals required for the survival and differentiation of immature T cells, referred to as thymocytes. Medullary TECs (mTECs) play a critical role in preventing autoimmunity because they have the unique ability to express peptides found in other organs throughout the body. Presentation of self-peptides to thymocytes causes deletion of cells that express high affinity self-reactive receptors. Numerous studies have established that a major signaling pathway mediated by NFκB family members is indispensable for mTEC development. However, whether other signaling pathways are also required has remained an open question. Here, we use gain- and loss-of-function genetic approaches to demonstrate that another pathway, mediated by Stat3 signaling, plays an important role in mTEC development and homeostasis. We show that constitutive Stat3 activation enhances the survival of immature mTECs and bypasses the requirement for thymocyte-derived signals in medullary region formation. In contrast, Stat3 depletion reduces mTEC cellularity and impairs medullary region formation.

## Introduction

The thymus provides a unique stromal microenvironment that is indispensable for the development of T cells that are both self-restricted and self-tolerant. Thymic epithelial cells (TECs) comprise the major component of the three-dimensional thymic stromal network, which also includes fibroblasts, dendritic cells (DCs) and endothelial cells [1–3]. TECs produce cytokines and cell surface ligands that promote thymocyte survival, growth and differentiation as well as chemokines that direct thymocyte migration into distinct cortical and medullary zones that contain phenotypically and functionally distinct TEC subsets [1, 2, 4]. Cortical TECs (cTECs) express Delta-like 4 ligands that engage Notch receptors on immature CD4^-^CD8^-^ double negative (DN) thymocytes to promote their maturation to the CD4^+^CD8^+^ double positive (DP) stage [5, 6]. In addition, cTECs present self-peptide/MHC complexes that instigate positive selection of DP thymocytes with low avidity αβTCRs. Positively selected thymocytes differentiate to the CD4^+^CD8^-^ or CD4^-^CD8^+^ single positive (SP) lineage and migrate into the medulla where they reside for several days and are subjected to negative selection before export to the periphery [7–15].

Medullary TECs (mTECs) express a wide range of otherwise tissue-restricted antigens (TRAs) due, in part, to expression of the nuclear protein AutoImmune REgulator (AIRE) [16, 17]. TRAs may be presented to SP thymocytes directly by expression on mTECs or indirectly by transfer from mTECs to DCs [18–22]. In either case, the unique ability of mTECs to express TRAs is essential for deletion of SP thymocytes bearing TCRs with high avidity for self-peptides, thereby establishing central tolerance [7, 23]. In addition, mTECs are required to support the development of Foxp3^+^ T regulatory cells (Tregs), which function in the periphery to restrain immune responsiveness of self-reactive and potentially autoimmune T cells [24, 25]. The essential contribution of mTECs in preventing the emergence and/or function of self-reactive peripheral T cells is evident from the autoimmune manifestations that occur in the absence of a functional mTEC compartment [26–29].

Considering the indispensable role that mTECs play in establishing central tolerance, the mechanisms regulating their differentiation, organization and maintenance are of considerable interest. It is well established that crosstalk between thymocytes and TECs is required for development and expansion of both cell types [30]. During fetal thymus development, lymphoid tissue inducer cells and γδTCR^+^ thymocytes provide signals that initiate mTEC differentiation to generate small medullary islets [31, 32]. In the postnatal period, signals from SP thymocytes play a nonredundant role in expanding the mTEC compartment to establish an organized and functional medullary region [33, 34]. When thymocyte maturation is arrested at the DP stage, as occurs in TCRα deficient and MHC class I/MHC class II double deficient mice, small, hypocellular medullary foci are produced [35, 36]. Numerous investigations have demonstrated that SP thymocytes promote mTEC development via activation of the NFκB signaling pathway. SP thymocytes express tumor necrosis factor superfamily (TNFSF) ligands such as CD40 ligand (CD40L), receptor activator of NFκB ligand (RANKL), lymphotoxin-α (LTα) and LTβ that bind corresponding TNFSF receptors on mTECs to activate NFκB signaling. In the absence of TNFSF ligands and/or receptors, mTEC differentiation and organization are severely impaired resulting in multi-organ autoimmunity [26, 28, 32, 37–41]. Similarly, deletion of RelB, TNF receptor-associated factor 6 (TRAF6) and other components in the noncanonical or canonical NFκB pathways blocks mTEC differentiation leading to the emergence of autoimmune symptoms [27, 29, 42–44]. Furthermore, a recent study showed that signals from SP thymocytes are required to overcome TRAF3 mediated inhibition of noncanonical NFκB signaling [45].

Although it is well established that NFκB signaling is essential for mTEC development and that multiple TNFSF receptor/ligand combinations are involved in mTEC differentiation and expansion, the nature and severity of medullary defects vary depending on the particular component of the NFκB pathway that is inactivated. These findings imply that additional signaling pathways may contribute to the regulation of mTEC development and maintenance. Here we report that Signal transducer and activator of transcription 3 (Stat3) is required for optimal medullary region formation and mTEC homeostasis. Stat3 is a transcription factor that regulates diverse cellular processes including survival, proliferation and differentiation in a cell type specific manner [46, 47]. We used Stat3 gain-of-function (GOF) and loss-of-function (LOF) genetic models to interrogate the contribution of Stat3 signaling to mTEC development and homeostasis. The data show that targeting a constitutively active Stat3 transgene to the mTEC compartment increases mTEC cellularity and expands the thymic medulla. Conversely, conditional deletion of Stat3 in mTECs reduces mTEC cellularity and impairs medullary region formation. In addition, we show that Stat3 activation is required for mTEC survival, but not for proliferation and may also play a role in differentiation. The precisely contrasting outcomes imposed by enforcing or preventing Stat3-mediated signaling in mTECs reveal that Stat3 activation plays a key role in medullary region formation and mTEC maintenance.

## Results

### Selective expansion of the medullary region in K5.Stat3C transgenic thymi

**Fig 1:**
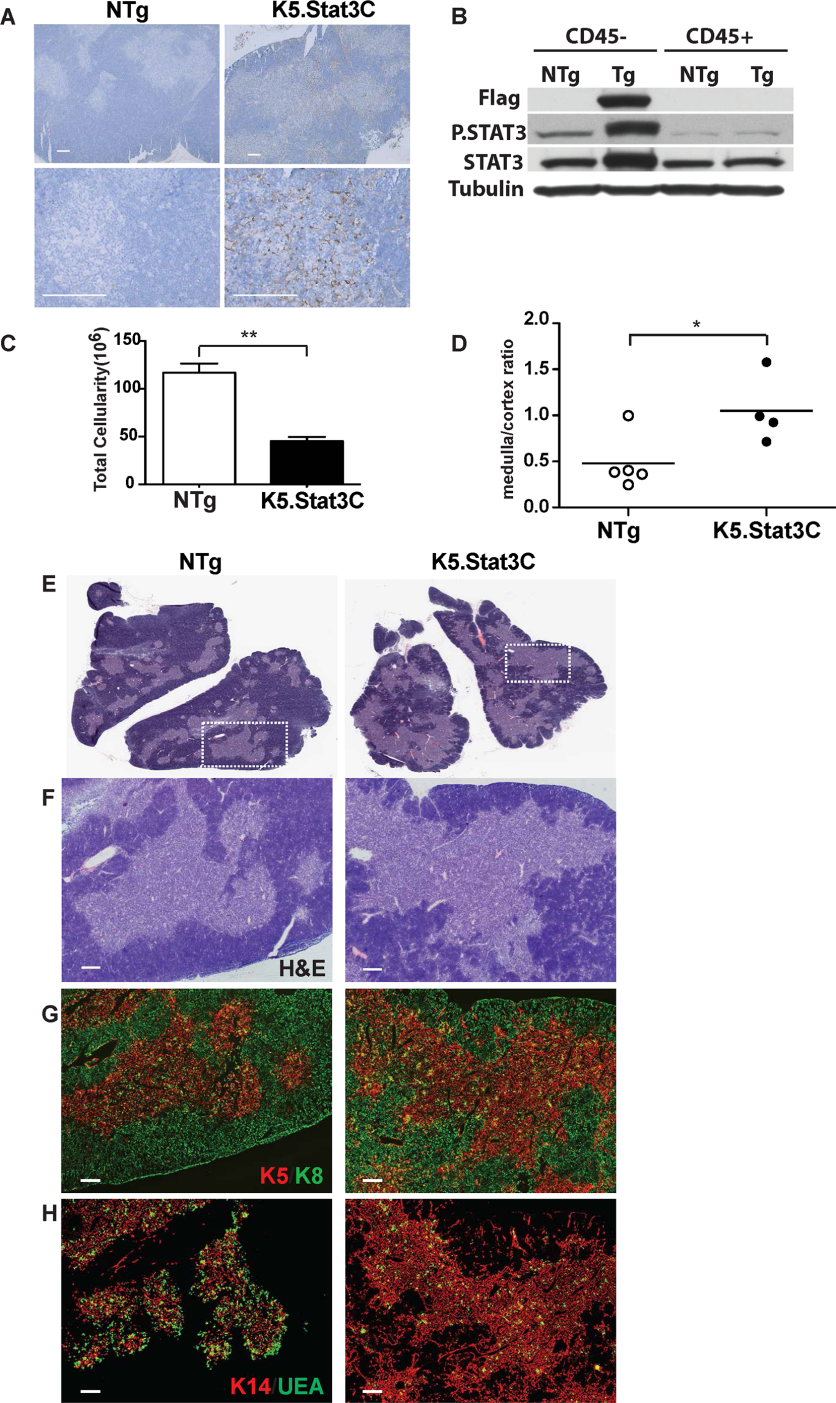
Reduced thymus cellularity and increased medulla to cortex ratio in K5.Stat3C transgenic mice. **(A)** IHC staining showing expression of the Flag tag in medullary regions of K5.Stat3C transgenic thymi. **(B)** Western blots show the Flag tag is expressed in CD45- stromal cells, but not in CD45+ hematopoietic cells. Elevated levels of phosphorylated Stat3 are also restricted to stromal cells. **(C)** Total thymus cellularity is reduced in K5.Stat3C transgenic (n = 9 for all) compared to control NTg littermates. **P<0.005 (Student’s paired *t*-test) **(D)** Medulla/cortex ratio in K5.Stat3C (n = 4) and control (n = 5) thymi determined by morphometric analysis of Aperio scanned H&E stained sections. *P<0.05 (Student’s paired *t*-test) (**E-H**) H&E and immunofluorescence (IF) stains of serial frozen thymus sections. **(E)** Low power image of H&E stained thymus section; the area demarcated by the dotted white is magnified in f-g. **(F)** H&E stain; **(G)** K5 and K8; **(H)** K14 and UEA-1. Scale bar: 200μm. Images are representative of sections from at least 5 NTg control and 5 K5.Stat3C transgenic thymi.

The constitutively active Stat3 transgene (K5.Stat3C) is highly expressed in the thymic medulla and in scattered cells throughout the cortex as shown by immunohistochemical (IHC) staining for the Flag epitope tag (**Fig 1A**). Western blot analysis showed elevated levels of total and phosphorylated Stat3 in extracts from CD45 negative, but not CD45 positive cells, confirming that K5.Stat3C transgene expression is restricted to thymic stromal cells (**Fig 1B**). Overall thymus cellularity was reduced by ~60% in K5.Stat3C transgenic mice compared to nontransgenic (NTg) littermates, hereafter referred to as NTg controls (**Fig 1C**). Hematoxylin and eosin (H&E) stained sections revealed a striking expansion of the medullary region and corresponding reduction of the cortical region in K5.Stat3C transgenic thymi (**Fig 1E and 1F**). Quantitative analysis of Aperio scanned slides confirmed the increased medullary to cortical ratio (**Fig 1D**). IHC staining revealed that the K5.Stat3C transgenic thymus contained typical cTEC and mTEC subsets. Thus, K8^hi^K5^-^K14^-^UEA-1^-^ cTECs were present in the cortex, whereas the medulla contained both K8^lo^K5^+^K14^+^UEA-1^-^ and K8^hi^K5^-^K14^-^UEA-1^+^ mTEC subsets (**Fig 1G and 1H**) [48, 49]. In contrast to the well-demarcated corticomedullary junction (CMJ) in NTg controls, K14+ mTECs in K5.Stat3C thymi extended beyond the CMJ into the cortex demonstrating that medullary expansion was associated with disruption of the typically circumscribed boundary at the CMJ.

### Expression of the K5.Stat3C transgene in RAG-2^-/-^ mice generates medullary region formation in the absence of thymocyte-derived signals

**Fig 2:**
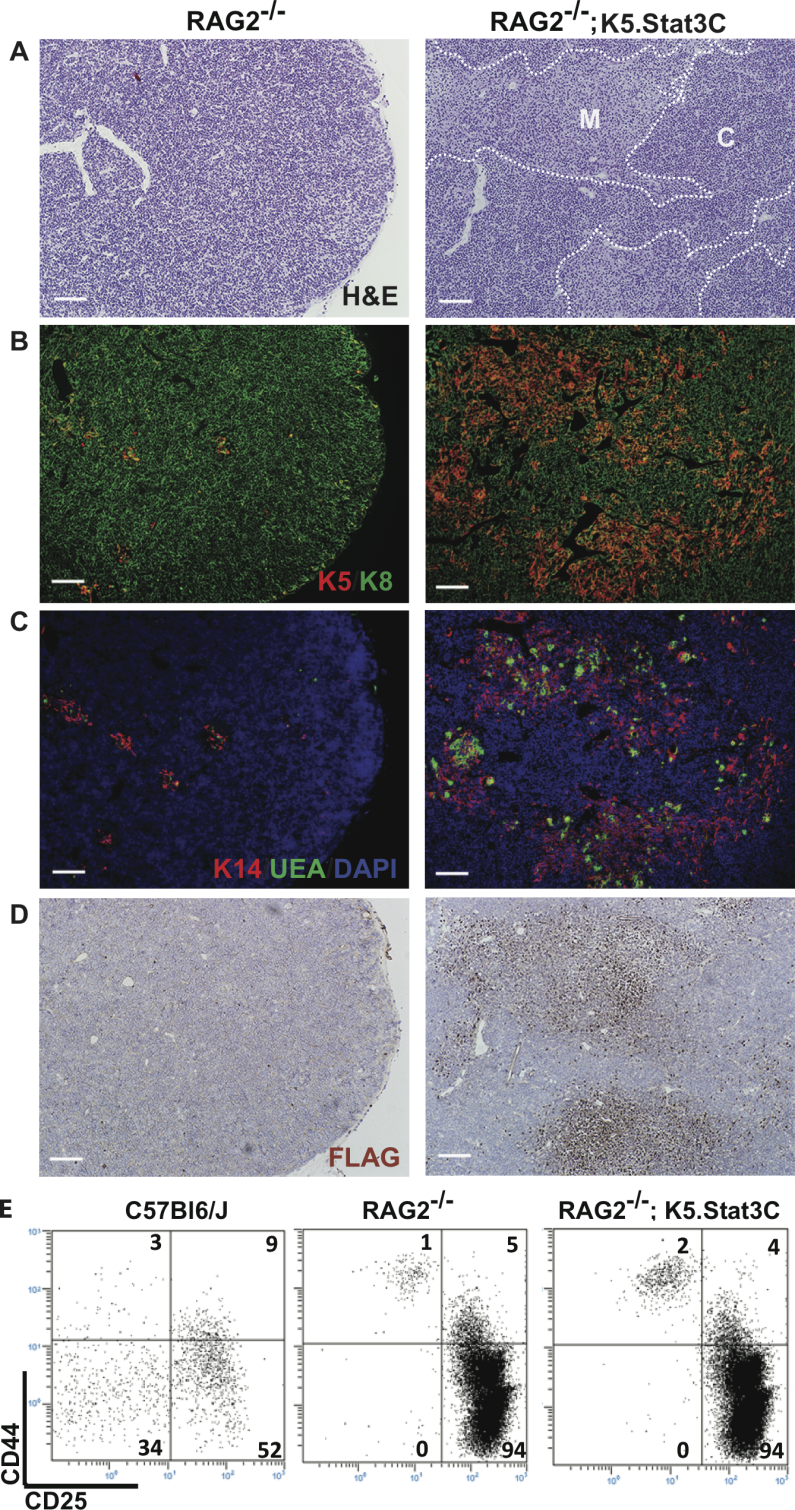
K5.Stat3C expression generates medullary regions in RAG-2^-/-^ thymi. (a-d) Serial frozen sections of RAG-2^-/-^ and RAG-2^-/-^;K5.Stat3C thymi. **(A)** H&E stain shows medullary region expansion in RAG-2^-/-^;K5.Stat3C thymi. The dotted line separates cortex (C) and medulla (M). (b-d) IHC staining showing expression of **(B)** K5 and K8; **(C)** K14, UEA-1 and DAPI; **(D)** Flag epitope. Data are representative of at least 5 mice of each genotype. **(E**) FACS analysis of CD4-CD8- thymocyte subsets identified, by CD25 and CD44 expression, from wildtype, RAG-2^-/-^ and RAG-2^-/-^;K5.Stat3C mice. The percentage of cells in each subset is shown on the FACS plots. FACS data are representative of 3 mice from each genotype. Scale bar: 200μm.

We considered that the thymus hypoplasia and increased medullary/cortical ratio could be a consequence of stress-induced DP thymocyte depletion particularly since K5.Stat3C transgenic mice develop psoriasis-like skin lesions [50]. To resolve this issue, we introduced the K5.Stat3C transgene into RAG-2^-/-^ mice in which thymocyte development is arrested at the CD44^-^CD25^+^ DN3 stage. Medullary region development is severely compromised in the RAG-2^-/-^ thymus due to the absence of positively selected thymocytes, which normally drive mTEC expansion [38, 39, 51]. The small medullary foci in RAG-2^-/-^ thymi were populated with sparse clusters of K5^+^ K14^+^ mTECs and rare UEA-1 binding cells (**Fig 2A, 2B and 2C**). In contrast, RAG-2^-/-^;K5.Stat3C thymi contained large medullary regions containing abundant K5^+^K14^+^ and UEA-1 binding mTEC subsets. Furthermore, the pattern of Flag epitope expression indicated that the K5.Stat3C transgene is highly expressed throughout the expanded medullary regions, as well as in scattered cortical cells (**Fig 2D)**. DAPI negative areas were apparent in the vicinity of the enlarged medullary regions in RAG-2^-/-^;K5.Stat3C thymi. Co-staining for K14 and VE-cadherin (S1 Fig) revealed that most of these regions are blood vessels, in accord with the concept that thymic vasculature plays a role in medullary compartment organization [52]. However, a few K14 bounded cyst-like structures were also present, consistent with active expansion and remodeling of this compartment.

Flow cytometric analysis verified that thymocyte differentiation remains arrested at the DN3 stage in RAG-2^-/-^ mice that express the K5.Stat3C transgene (**Fig 2E**). These data show that the medullary region expansion observed in RAG-2 sufficient K5.Stat3C thymi is not a trivial consequence of stress-induced cortical thinning. Rather, the results indicate that constitutive activation of Stat3 in RAG-2^-/-^ TECs bypasses the requirement for signals from positively selected thymocytes and independently drives medullary region formation.

### The number of mTECs is increased in K5.Stat3C thymi

**Fig 3:**
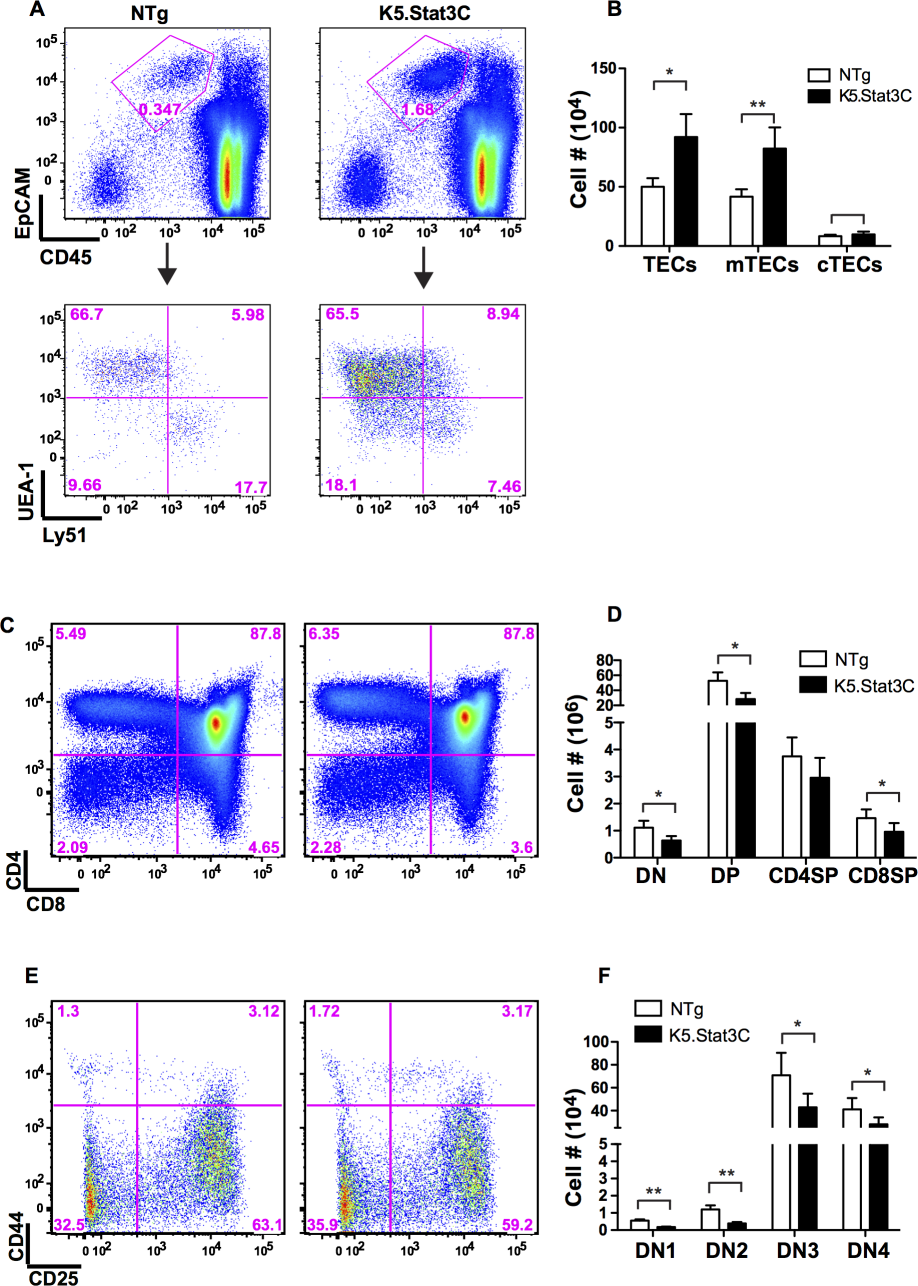
The number of mTECs, but not cTECs, is increased in K5.Stat3C transgenic thymi. **(A)** Representative FACS plots show the frequency of total TECs, which are CD45-EpCAM+ (top row), as well as UEA-1-Ly51+ cTECs and UEA-1+Ly51- mTECs (bottom row) in K5.Stat3C and control thymi. The percentage of cells in each gate or quadrant is shown. **(B)** Bar graph (mean ± SD) shows cellularity of total TECs, cTECs and mTECs (n = 8 each) **(C)** Representative FACS plots of major thymocyte subsets defined by CD4 and CD8 expression. **(D)** Bar graph shows cell number (mean ± SD) of each subset in K5.Stat3C and control thymi (n = 8 each) **(E)** Representative FACS plots of DN subsets. **(F)** Bar graph shows cell number in each DN subset. *P<0.05 and **P<0.005 (Student’s paired *t*-test)

K5.Stat3C transgenic and NTg control thymi were enzymatically dissociated for flow cytometric analysis of TECs, which were identified as CD45^-^ MHCII^+^ EpCAM^+^ cells. Both the percentage and absolute number of total TECs were increased in K5.Stat3C compared to control thymi (**Fig 3A and 3B**). The mTEC and cTEC subsets were distinguished by UEA-1 binding and Ly51 expression [53–55]. Despite a decrease in the frequency of UEA-1^-^ Ly51^+^ cTECs (**Fig 3A**), the absolute number of cTECs was comparable in K5.Stat3C and control thymi (**Fig 3B**). In contrast, there was a 2-fold increase in the number of UEA-1^+^ Ly51^-^ mTECs in K5.Stat3C thymi. Expansion of the mTEC, but not the cTEC compartment, is consistent with the increased medullary/cortical ratio observed in H&E stained sections of K5.Stat3C thymi (**Fig 1**). However, the selective medullary expansion was not due to mTEC restricted transgene expression. Consistent with IHC stains showing Flag epitope positive cells in the cortex and medulla (**Fig 1A**), qRT-PCR analysis demonstrated that the Stat3C transgene is expressed in cTECs and mTECs (S2A Fig). Similarly, FACS analysis revealed increased levels of the active phosphorylated form of Stat3 protein in transgenic cTECs and mTECs compared to their NTg counterparts (S2B Fig).

We asked whether the expanded medullary regions and increased number of mTECs in K5.Stat3C thymi affected the cellularity of immature and mature thymocyte subsets. Flow cytometric analysis showed that transgenic and NTg control thymi contained comparable percentages of thymocytes in the major subsets defined by CD4 and CD8 expression, as well as in the DN subsets defined by CD44 and CD25 expression (**Fig 3C and 3E**). However, K5.Stat3C transgenic thymi contained fewer thymocytes in each subset including the T cell progenitor containing DN1 subset (**Fig 3D and 3F**). The factors responsible for decreased thymocyte cellularity are presently unknown. However, given the reduction in DN1 numbers, we speculate that indirect effects on availability and/or function of microenvironmental niches that support thymocyte progenitors could play a role [56, 57]. Although cellularity and organization of the cTEC compartment appear normal, it is possible that defects in a minor cTEC component could account for the reduced thymocyte cellularity phenotype.

### An immature MHCII^lo^CD80^lo^ mTEC subset is selectively expanded in K5.Stat3C thymi

**Fig 4:**
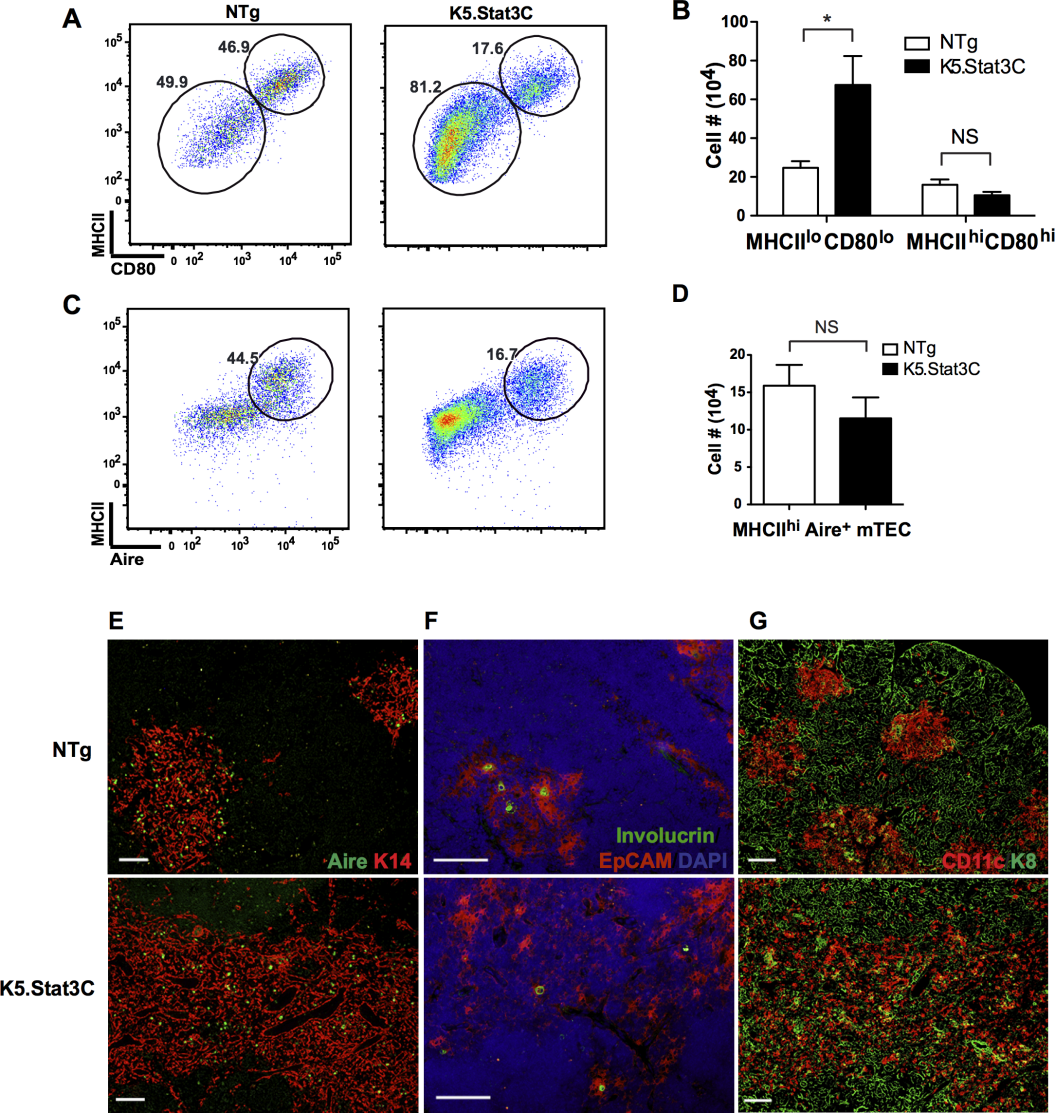
MHCII^lo^CD80^lo^ mTECs are selectively expanded in K5.Stat3C transgenic thymi. **(A)** Representative FACS plots show percentage of MHCII^lo^CD80^lo^ and MHCII^hi^CD80^hi^ mTECs. **(B)** Bar graph shows number (mean ± SD) of MHCII^lo^ and MHCII^hi^ mTECs in K5.Stat3C and control thymi (n = 5 each). **(C)** FACS plots show percentage of MHCII^hi^Aire^+^ mTECs. **(D)** Bar graph shows number (mean ± SD) of MHCII^hi^Aire^+^ mTECs (n = 5). **(E)** IHC stains show abundant K14+ and Aire+ mTECs in K5.Stat3C thymi. Scale bar: 200μm. **(F)** The frequency of involucrin+ mTECs is not increased in K5.Stat3C thymi. Scale bar: 100μm. **(G)** IHC stains show K8 expressing cTECs and CD11c expressing DCs. Scale bar: 200μm. *P<0.05 (Student’s paired *t*-test).

Previous studies have shown that functionally mature Aire positive mTECs expressing higher levels of MHC class II and CD80 arise from Aire negative precursors that express relatively low levels of MHC class II and CD80 [32, 58, 59]. Interestingly, there is a marked increase in the frequency and number of immature MHCII^lo^CD80^lo^ mTECs in K5.Stat3C compared to NTg control thymi (**Fig 4A and 4B)**. Comparable results were obtained when CD40 rather than CD80 expression was assessed (**S3 Fig**).

That the expanded MHCII^lo^ mTEC population does not express high levels of Aire is consistent with the notion that this subset consists of immature mTECs. However, Aire expression may be downregulated at later stages of TEC differentiation. A recent investigation of mTEC subset lineage relationships showed that Aire^+^CD80^hi^ mTECs can give rise to Aire negative mTECs in which CD80 is downregulated to intermediate expression levels [60]. Moreover, signals from positively selected thymocytes can induce Aire^+^ mTECs to advance to an Aire^-^ differentiation stage characterized by expression of involucrin, a marker of terminally differentiated keratinocytes [61]. Since Aire expression may be downregulated in differentiated mTECs, we asked if the expanded subset of MHC II^lo^CD80^lo^ Aire^-^ mTECs in K5.Stat3C transgenic mice might represent a terminal stage of mTEC differentiation as opposed to an immature progenitor stage. We conclude that this is not the case because involucrin positive TECs were not increased in K5.Stat3C thymi (**Fig 4F**). Moreover, the expanded subset of MHCII^lo^ transgenic TECs expresses low to negative, rather than the intermediate levels of CD80 previously described for mature Aire negative TECs [60] **(Fig 4A**). These data indicate that the increased number of mTECs in K5.Stat3C thymi is a consequence of expansion of the immature MHCII^lo^CD80^lo^Aire^-^ mTEC subset.

Similar to the thymic phenotype in RAG sufficient K5.Stat3C mice, the number of mTECs was significantly increased in RAG-2^-/-^;K5.Stat3C thymi, primarily due to an ~15 fold expansion of the MHCII^lo^ mTEC subset (S4A Fig). Cellularity in the MHCII^hi^ mTEC compartment also increased, albeit to a lesser extent (~6 fold) than that of the MHCII^lo^ subset. Importantly, IHC staining (S4B Fig) revealed Aire+ cells in the expanded RAG-2^-/-^;K5.Stat3C medullary regions demonstrating that at least some cells in the expanded immature mTEC subset differentiate to a MHCII^hi^ Aire+ stage in the absence of thymocyte-derived signals.

### TRA expression is not altered and autoimmune symptoms are not detected in K5.Stat3C mice

Expression of TRAs by mTECs is essential for negative selection of autoreactive thymocytes and establishment of central tolerance. Despite the lower percentage of Aire^+^ mTECs (**Fig 4C**) in K5.Stat3C thymi, there was no significant difference in the total number of transgenic MHCII^hi^ Aire^+^ mTECs compared to NTg littermate controls (**Fig 4D**) and Aire expressing TECs were appropriately localized in the medulla (**Fig 4E**). Furthermore, qRT-PCR analysis of sorted MHCII^hi^ mTECs verified comparable expression of *Aire* mRNA as well as Aire-dependent and Aire-independent TRAs in K5.Stat3C and control thymi (**S5 Fig**). Dendritic cells also play an important role in deleting autoreactive thymocytes by cross-presenting TRAs [19, 22]. Mature mTECs secrete XCL1, a chemokine that is essential for proper accumulation of thymic dendritic cells in the medulla [62]. The presence of CD11c^+^ dendritic cells at the corticomedullary junction and throughout the medullary region of K5.Stat3C thymi (**Fig 4G**) suggests that transgenic mTECs function normally to recruit dendritic cells. Overt autoimmune symptoms were not found in K5.Stat3C transgenic mice up to 9 months of age. Histological examination of tissue sections revealed no evidence of lymphocytic infiltrations in kidney, lacrimal gland, liver or pancreas (**S5D Fig**). Furthermore, autoantibodies were not detected when RAG2^-/-^ tissue sections were incubated with serum from K5.Stat3C mice (**S6 Fig**). The absence of autoimmune symptoms is consistent with expression of TRAs by phenotypically mature mTECs and the presence of DCs in K5.Stat3C transgenic thymi.

### The K5.Stat3C transgene enhances mTEC survival but not proliferation

**Fig 5:**
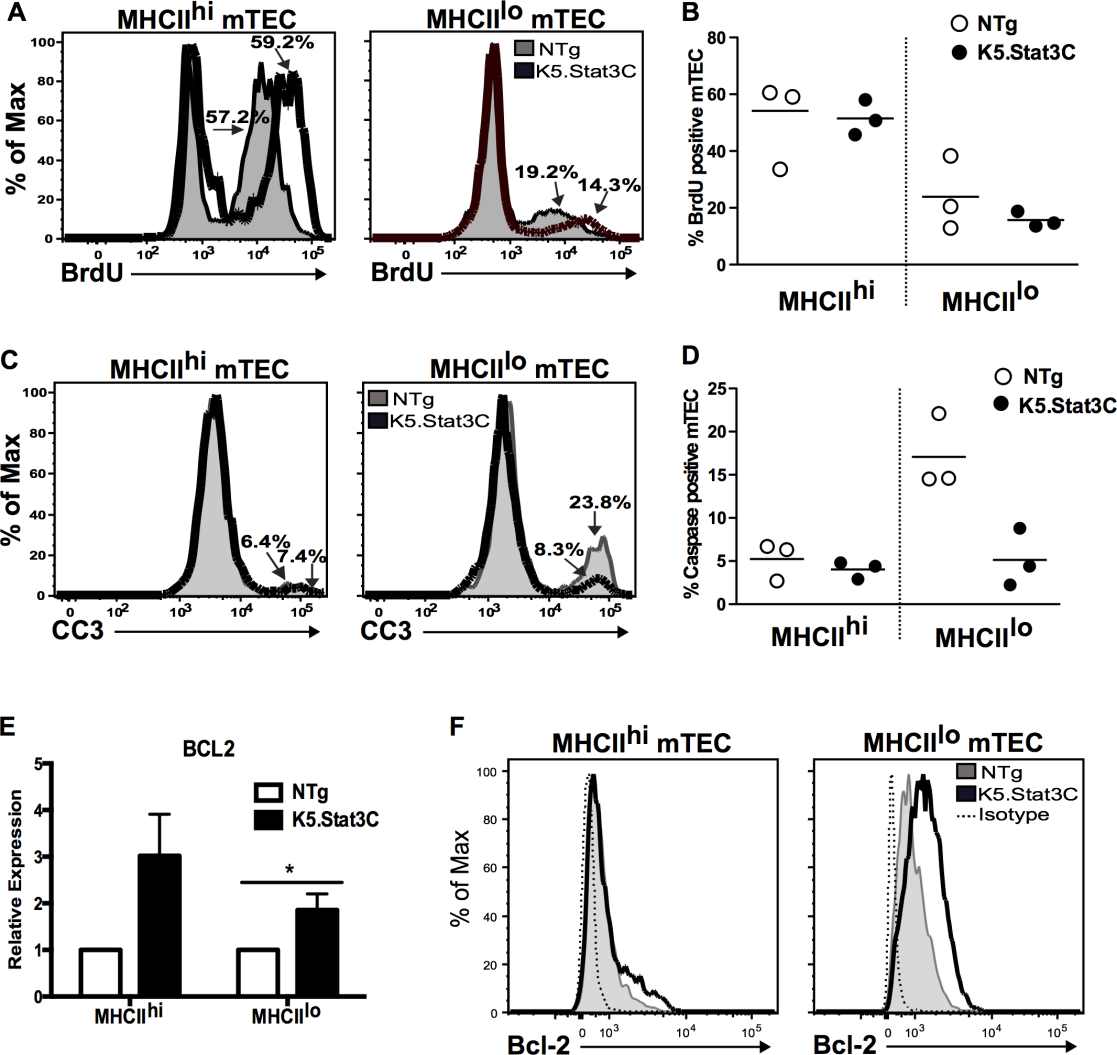
Expression of K5.Stat3C enhances MHCII^lo^CD80^lo^ mTEC survival, but not proliferation. **(A)** Representative FACS histograms showing frequency of BrdU positive cells in MHCII^hi^ and MHCIl^lo^ mTEC subsets from K5.Stat3C (thick black line) and control (shaded) thymi. **(B)** Scatter graphs showing percentage of BrdU positive cells. **(C)** Representative FACS histograms showing frequency of cleaved caspase 3 (CC3) positive cells in MHCII^hi^ and MHCIl^lo^ mTEC subsets from K5.Stat3C (thick black line) and control (shaded) thymi. **(D)** Scatter graphs showing percentage of CC3 positive cells. **(E)** Quantitative RT-PCR analysis of *Bcl-2* expression in MHCII^hi^ and MHCIl^lo^ mTECs. Relative expression of *bcl-2* mRNA was normalized using α-tubulin mRNA and the NTg MHCIl^hi^ mTEC control was set at 1. Bar graph shows mean ± SEM of four independent experiments with duplicate or triplicate samples in each experiment. *P<0.05 (Student’s unpaired *t*-test). **(F)** Representative FACS histograms showing Bcl-2 expression in MHCII^hi^ and MHCIl^lo^ mTEC subsets from K5.Stat3C (thick black line) and control (shaded) thymi.

Stat3 activation regulates a diverse set of cellular processes, including proliferation and apoptosis, in a cell type specific manner [63]. Both mature and immature mTECs contain a proliferative compartment, although mature mTECs have a higher frequency of cycling cells [58, 59, 64]. We analyzed BrdU incorporation to determine if expansion of the MHCII^lo^CD80^lo^ subset in K5.Stat3C thymi is a function of increased proliferative activity. Consistent with previous reports, we found a higher percentage of cycling cells in MHCII^hi^ compared to MHCII^lo^ mTECs in control NTg thymi [58, 59]. There was no increase in the frequency of BrdU positive cells in MHCII^lo^ or MHCII^hi^ mTEC subsets from K5.Stat3C thymi indicating that expansion of the immature MHCII^lo^ subset is not attributable to increased proliferation (**Fig 5A and 5B**).

To assess survival, we determined the frequency of apoptotic cells based on expression of cleaved caspase-3. The percentage of cleaved caspase-3 positive MHCII^hi^ mTECs was comparable in K5.Stat3C and control thymi (**Fig 5C**). However, there was a marked reduction in the percentage of cleaved caspase-3 positive cells in MHCII^lo^ mTECs from K5.Stat3C thymi suggesting that enhanced survival contributes to expansion of this subset (**Fig 5C and 5D**). This notion was supported by qRT-PCR analysis showing that compared to NTg controls, K5.Stat3C MHCII^lo^ mTECs express significantly increased levels of the anti-apoptotic gene *bcl-2*, which is a downstream target of the Stat3 signaling pathway (**Fig 5E**) [65, 66]. Increased *bcl-2* expression was also found in the MHCII^hi^ mTEC subset from K5.Stat3C compared to NTg thymi; however, statistical significance was not achieved due to greater variability (*P* = 0.065). Analogous results were obtained when levels of Bcl-2 protein were assessed by FACS analysis (**Fig 5F**). A clear increase in Bcl-2 was apparent in MHCII^lo^ mTECs, whereas only a modest increase was observed in the MHCII^hi^ subset.

### Conditional deletion of Stat3 disrupts medullary region architecture

The results obtained in K5.Stat3C mice demonstrate that constitutive activation of Stat3 promotes the survival and accumulation of MHCII^lo^ mTECs. However, these data do not demonstrate whether Stat3 activity is indispensable for mTEC survival and/or differentiation. An earlier report found that Stat3 is not essential for proper mTEC development and medullary formation [67]. In that investigation, a K5 promoter driven Cre transgene was introduced into *Stat3*^*fl/fl*^ or *Stat3*^*fl/-*^ mice to delete Stat3 in K5 positive TECs. The investigators reported that conditional deletion of Stat3 generated a hypoplastic thymus phenotype that progressed with age and was associated with loss of DP thymocytes, cortical atrophy and misalignment of cTECs [67]. However, the mTEC compartment appeared morphologically normal.

**Fig 6:**
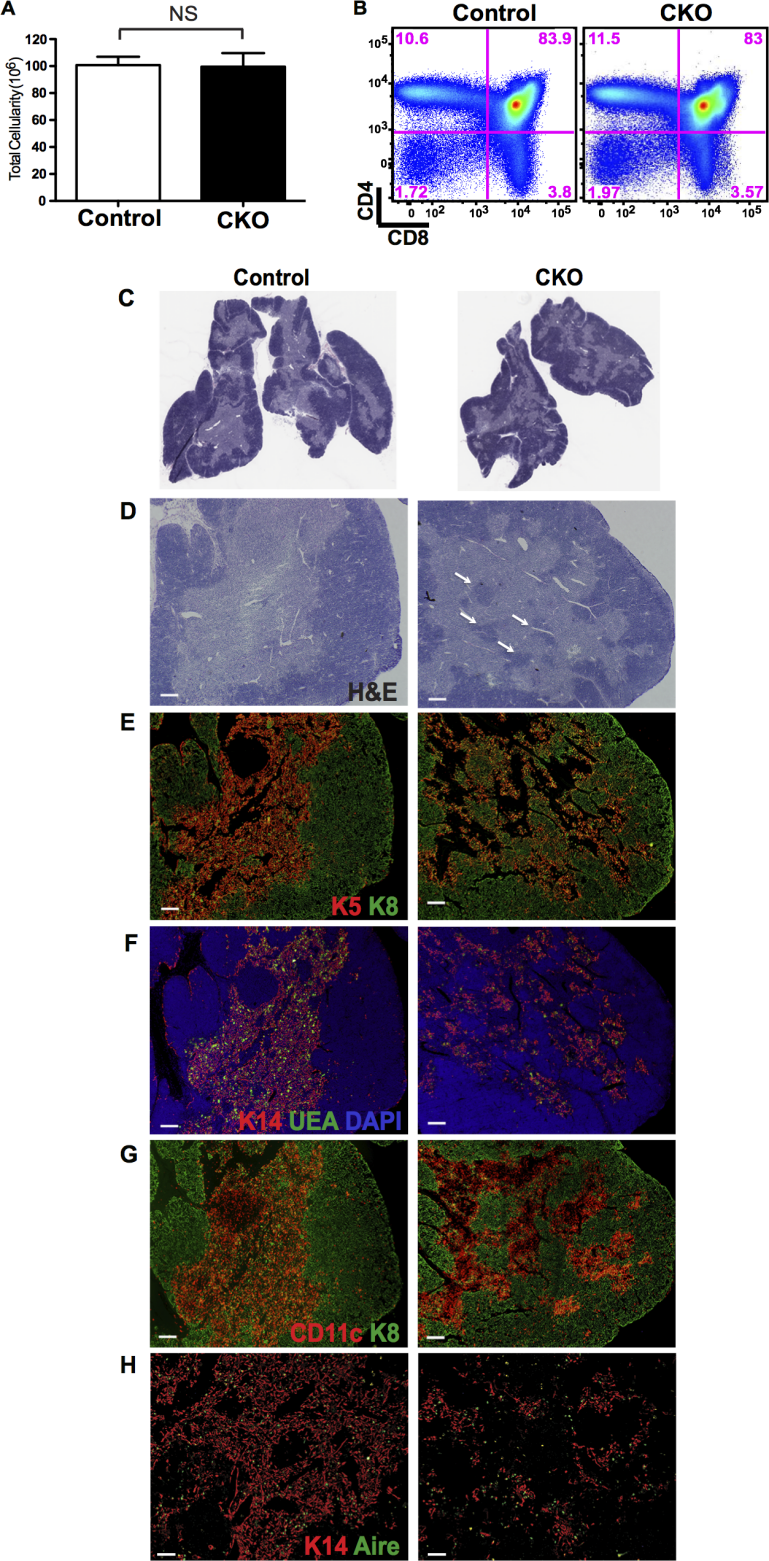
Depletion of Stat3 in the TEC compartment disrupts medullary architecture. **(A)** Thymus cellularity is comparable in Stat3 CKO (n = 9 for both) and controls. (p = 0.9960, Unpaired Student’s T test) **(B)** FACS plots show comparable frequency of major thymocyte subsets defined by CD4 and CD8 expression. The percentage of cells in each quadrant is shown. Results are representative of 5 mice of each genotype. **(C)** Low power image of H&E stained frozen thymic sections. **(D-H)** IHC stains on frozen sections of CKO and control thymi **(D)** H&E **(E)** K5 and K8 **(F)** K14, UEA-1 and DAPI **(G)** CD11c and K8 **(H)** K14 and Aire. All images are serial sections with the exception of the control section in H. Results are representative of at least 5 thymi of each genotype. Scale bar: 200μm.

Using the same strategy, we introduced a *K5*.*Cre* transgene into mice that were homozygous for the same floxed *Stat3* allele to examine the effect of Stat3 deletion in mTECs. PCR analysis showed efficient deletion of floxed *Stat3* alleles in total TECs as well as in sorted cTECs and mTEC subsets **(S7A and S7B Fig)**. Furthermore, FACS analysis confirmed extensive depletion of Stat3 protein in cTEC and mTEC subsets from conditional knock out compared to control thymi **(S7C Fig)**. In contrast to the earlier study [67], we did not observe either thymus hypoplasia nor depletion of DP thymocytes in 6–12 week old *K5*.*Cre;Stat3*^*fl/fl*^ mice, hereafter referred to as Stat3 conditional knock out (Stat3 CKO) mice. Instead, the number and percentage of immature and mature thymocytes were comparable in CKO and littermate controls (**Figs 6A, 6B** and **S9**). Quantitative analysis of Aperio scanned H&E stained sections showed no alteration in the cortical/medullary ratio of Stat3 CKO compared to control thymi (**S9 Fig**). Nevertheless, striking abnormalities in medullary architecture and mTEC cellularity were obvious in the Stat3 CKO thymi. For example, there was an increase in medullary islets (**Fig 6C**) and small cortical foci were frequently embedded in the medulla (**Fig 6D**). The medullary regions in Stat3 CKO thymi appeared tattered and were sparsely populated by mTECs that were dispersed in irregular clusters, rather than uniformly distributed throughout the medulla as in control thymi. (**Fig 6E and 6F**. Despite the paucity of mTECs, the presence of both K5^+^K14^+^ and UEA-1 binding subsets suggests that Stat3 is not absolutely essential for mTEC development. The presence of Aire+ mTECs further indicated that mTEC maturation was at least partially intact (**Fig 6G**). In contrast to the relative scarcity of mTECs, CD11c+ dendritic cells were abundant in Stat3 CKO medullary regions indicating that recruitment of these cells was not impaired (**Fig 6H**).

### Conditional Stat3 deletion reduces the number of immature and mature mTEC subsets

**Fig 7:**
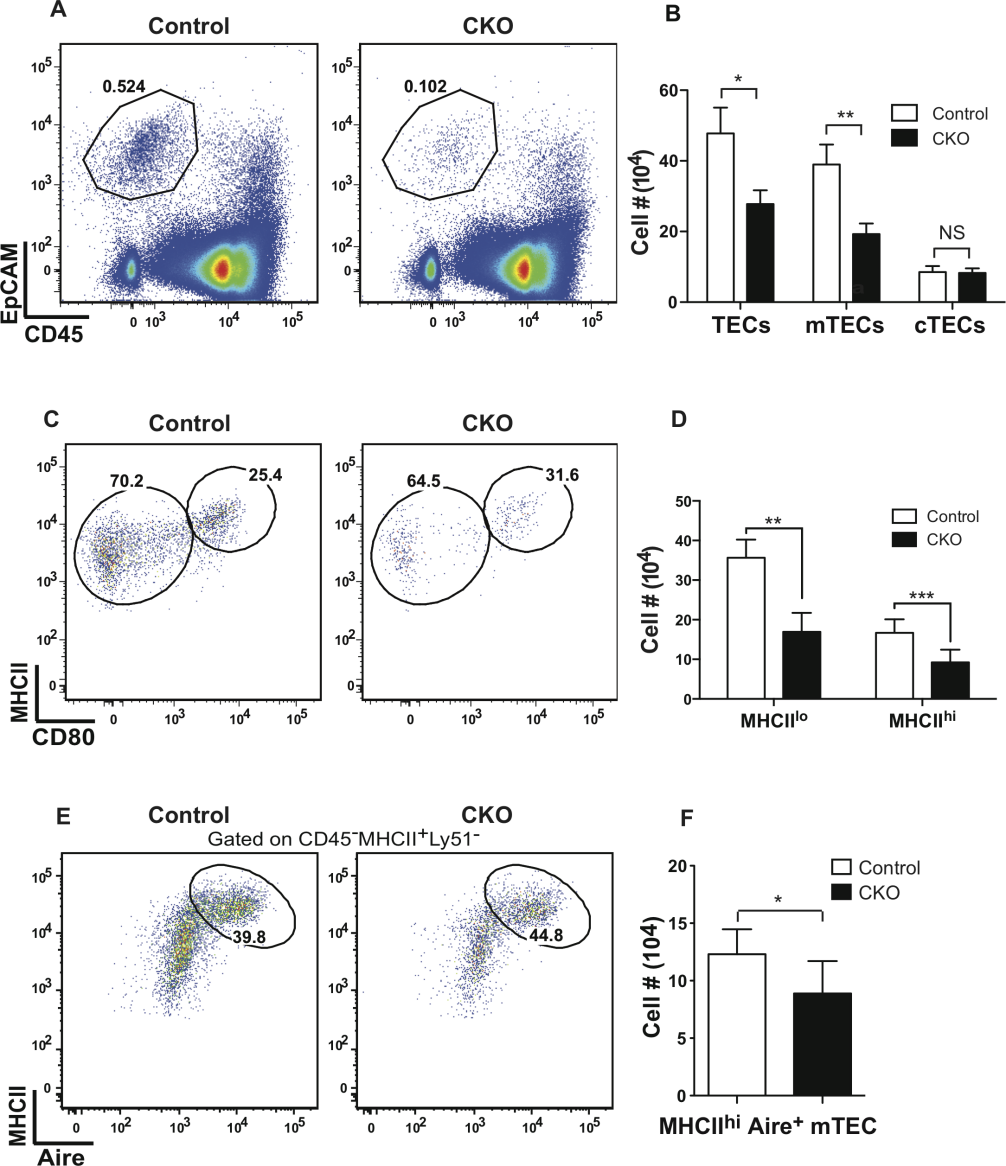
mTECs are selectively depleted in Stat3 CKO thymi. **(A)** Representative FACS plots show the frequency of CD45-EpCAM+ TECs in Stat3 CKO and control thymi. **(B)** Bar graph shows (mean ± SD) cellularity of total TECs, cTECs and mTECs (n = 5 for each) **(C)** Representative FACS plots show the frequency of MHCII^lo^CD80^lo^ and MHCII^hi^CD80^hi^ mTECs in Stat3 CKO and control thymi **(D)** Bar graph shows number (mean ± SD) of MHCII^lo^ and MHCII^hi^ mTECs (n = 5 for each). **(E)** Representative FACS plots show frequency of MHCII^hi^Aire^+^ mTECs. **(F)** Bar graph shows number (mean ± SD) of MHCII^hi^Aire^+^ mTECs (n = 5). *P<0.05 and **P<0.005 (Student’s paired *t*-test)

Consistent with the IHC data above, flow cytometric analysis showed that the percentage and number of TECs is decreased in Stat3 CKO thymi (**Fig 7A and 7B**). Loss of Stat3 selectively affected the mTEC subset as the number of cTECs was not altered. In stark contrast to the marked expansion of immature MHC II^lo^CD80^lo^ mTECs found in K5.Stat3C transgenic thymi **(Fig 3)**, this subset was depleted by ~50% in Stat3 CKO thymi (**Fig 7C and 7D**). The number of mature MHCII^hi^CD80^hi^ mTECs was also significantly decreased, a finding that is consistent with the precursor-progeny relationship between MHCII^lo^ and MHCII^hi^ mTEC subsets [32, 58, 59]. Despite reduced numbers, the percentage of Aire positive mTECs in Stat3 CKO thymi was comparable to that in controls (**Fig 7E and 7F**). Moreover, Stat3 CKO mTECs maintain the ability to express TRAs since expression levels of Aire-dependent and Aire-independent TRAs were comparable in FACS sorted MHCII^hi^ mTECs from Stat3 CKO and control thymi (**S10 Fig**). Histological examination of tissue sections from kidney, lacrimal gland, liver and pancreas revealed no evidence of lymphocytic infiltrations in Stat3 CKO mice up to 9 months of age (**S10 Fig**) Moreover, we found no evidence for the presence of auto-reactive antibodies after staining RAG2^-/-^ tissue sections with CKO serum (**S11 Fig**).

### Conditional Stat3 deletion does not alter the frequency of proliferating or cleaved caspase 3 positive mTECs, but does increase macrophage accumulation in medullary regions

**Fig 8:**
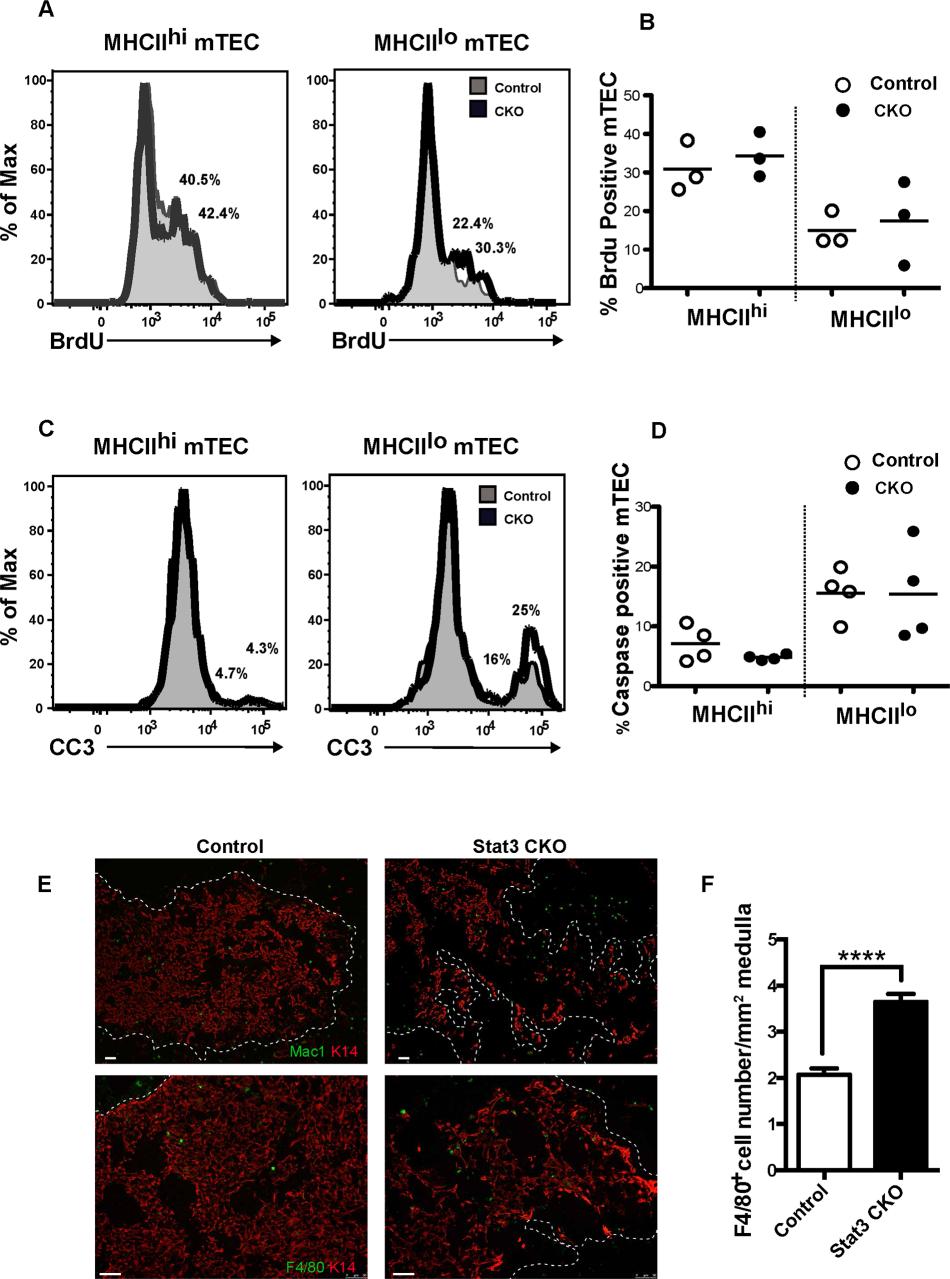
Effect of Stat3 depletion in mTEC proliferation and survival. **(A)** Representative FACS histograms showing frequency of BrdU positive cells in MHCII^hi^ and MHCIl^lo^ mTEC subsets from Stat3 CKO (thick black line) and control (shaded) thymi. **(B)** Scatter graphs showing percentage of BrdU positive cells. **(C)** Representative FACS histograms showing frequency of cleaved caspase 3 (CC3) positive cells in MHCII^hi^ and MHCIl^lo^ mTEC subsets from Stat3 CKO (thick black line) and control (shaded) thymi. **(D)** Scatter graphs showing percentage of CC3 positive cells. **(E)** Representative IHC stains for Mac1 and K14 (upper panels) and F4/80 and K14 (lower panels) showing an increased frequency of macrophages in the tattered Stat3 CKO medullary region. The dotted line shows the CMJ drawn based on DAPI stain. **(F)** Bar graph shows the number (mean ± SD) of F4/80+ cells per unit area (mm^2^) of medulla. Three medullary areas on each of three non-sequential sections (200μm apart) from two mice of each genotype were analyzed. ****P<0.0001 (Student’s paired *t*-test)

The frequency of proliferating mTECs in Stat3 CKO thymi was determined by FACS analysis of BrdU incorporation. We observed a comparable percentage of BrdU positive cells in MHCII^lo^ and MHCII^hi^ mTEC subsets from Stat3 CKO and control thymi (**Fig 8A and 8B**). Therefore, reduced mTEC proliferation does not account for depletion of the mTEC compartment in Stat3 CKO mice. Given the reduced percentage of cleaved caspase 3 positive cells observed in MHCII^lo^ mTECs from K5.Stat3C transgenic thymi (**Fig 5**), we speculated that the converse result would be obtained for MHCII^lo^ mTECs from Stat3 CKO mice. However, we did not find a reproducible increase in cleaved caspase 3 containing MHCII^lo^ mTECs from Stat3 CKO compared to control thymi (**Fig 8C and 8D).** Increased apoptosis is often difficult to detect as apoptotic cells are rapidly cleared from the thymus. Furthermore, fragile apoptotic TECs may be lost during the multiple rounds of enzymatic digestion required to obtain single cell suspensions for FACS analysis. Therefore, we analyzed CKO and control thymic sections for the presence of macrophages as a gauge of apoptotic activity. IHC staining using either Mac-1 or F4/80 antibody revealed an increase in macrophages within and adjacent to the disrupted medullary regions of Stat3 CKO compared to control thymi (**Fig 8E**). Quantifying the number of macrophages per unit area of medulla (**Fig 8F**) confirmed an increased accumulation of macrophages in the medullary regions of CKO thymi. These data support the notion that the reduction of mTECs in Stat3 CKO thymi is linked to increased cell death.

## Discussion

The reciprocal medullary phenotypes found in K5.Stat3C transgenic and Stat3 CKO mice demonstrate that Stat3 activation regulates mTEC development in the adult mouse thymus. Medullary region expansion and increased numbers of immature mTECs were present in K5.Stat3C transgenic thymi. Moreover, expression of the K5.Stat3C transgene in RAG2^-/-^ mice bypassed the requirement for thymocyte-derived signals in generating medullary regions containing functional mTEC subsets. In striking opposition, normal medullary architecture is disrupted and mTEC cellularity is reduced in Stat3 CKO thymi. The converse phenotypes in these GOF and LOF experimental models reveal that Stat3-mediated signaling is required for optimal medullary region formation and mTEC maintenance.

The presence of phosphorylated Stat3 in immature MHCII^lo^CD80^lo^ mTECs of steady-state thymi from young adult controls suggests that Stat3 signaling may be involved in thymus homeostasis. These results are consistent with a previous report that also implicated Stat3 in TEC homeostasis [68]. Thymocyte specific depletion of TRPM7, an ion channel and kinase dual function protein, partially blocked T cell development at the DN to DP transition. The corresponding progressive reduction in medullary region size was associated with a decrease in mTECs containing phosphorylated Stat3 [68]. Although a direct cause/effect relationship was not shown, these data were interpreted as suggesting that Stat3 activation is required for maintenance of the mTEC compartment [68]. While this report supports our present findings, the results we obtained for Stat3 CKO thymi differ starkly from another previous report in which deletion of Stat3 in K5 expressing TECs was found to alter the cTEC but not the mTEC compartment [67]. The reasons for this discrepancy are not clear but may involve differences in genetic background or in housing conditions that exacerbated stress leading to cortical thinning in the earlier report (64). In any case, our findings are entirely consistent with the results in an accompanying report from Satoh *et al*. who also found that Foxn1-Cre mediated deletion of Stat3 impairs medullary organization and reduces mTEC, but not cTEC cellularity.

It is well established that noncanonical NFκB activation is essential for mTEC differentiation and proliferation (reviewed in [3]). RelB deficient mice have small medullary foci that lack UEA-1 and Aire positive mTECs as well as dendritic cells [29, 69, 70]. The resulting defect in negative selection leads to extensive multi-organ lymphocytic infiltrates and autoimmunity. Similar autoimmune symptoms occur in the absence of signaling components upstream of RelB, such as NFκB inducing kinase (NIK) or IκB kinase α (IKKα) [27, 43]. Furthermore, the absence of mature mTECs in mice lacking TRAF6 demonstrates the importance of the canonical NFkB pathway [42]. The aberrant medullary phenotype in Stat3 CKO thymi is less severe than that caused by defects in the NFκB signaling pathway. The contracted mTEC compartment remaining in Stat3 CKO thymi likely accounts for the lack of overt autoimmune manifestations. Nevertheless, the present study and the accompanying report (Satoh *et al*.) clearly demonstrate that deleting Stat3 in TECs results in disorganized medullary regions and a severe reduction in mTEC cellularity. Thus, we conclude that, in addition to NFκB signaling, Stat3 activation plays an indispensable role in medullary region formation and mTEC homeostasis.

Whether the Stat3 and NFκB signaling pathways interface in mTECs may depend on the particular ligand/receptor pairs that are engaged. Multiple ligands including RANKL, CD40L and LTβ are upregulated on positively selected thymocytes and activate NFκB signaling upon interaction with corresponding receptors on mTECs [26, 38, 39, 71, 72] These ligand/receptor pairs differ in the extent to which they promote mTEC differentiation and/or proliferation. The absence of RANK or RANKL impairs mTEC proliferation and differentiation. As a consequence, the number of Aire positive mTECs is reduced, central tolerance is compromised and autoimmunity ensues [28, 32, 38]. The absence of CD40 or CD40L minimally affects mTEC cellularity or differentiation [28, 38, 71]; however, in the absence of both RANKL and CD40, medullary architecture is profoundly disrupted and the thymus is almost completely devoid of mTECs [28]. Furthermore, adoptively transferred spleen cells from RANKL and CD40 double deficient mice produced more extensive cellular infiltrates in liver and kidney of athymic nude recipients than spleen cells from mice deficient only in RANKL [28]. These data suggest that signaling pathways downstream of RANK and CD40 cooperate to promote mTEC differentiation and proliferation. Although the synergy between RANK and CD40 induced signals might arise from activation of both the canonical and noncanonical NFκB pathways, Stat3 activation may also be involved. In this regard, CD40 engagement has been shown to activate Stat3 signaling in B cells and cholangiocytes [73, 74]. Therefore, it is possible that CD40-mediated Stat3 activation is involved in the synergy between RANK and CD40 signaling in mTECs. TGFβ was recently shown to negatively regulate mTEC maturation [75]. It is possible that Stat3 activity affects mTECs by directly or indirectly modulating TGFβ expression or its downstream signaling pathways.

The increased number of mTECs in K5.Stat3C transgenic thymi is due to selective expansion of the MHCII^lo^CD80^lo^ Aire^-^ subset. The increase in MHC II^lo^CD80^lo^ mTECs is not a result of greater proliferation, but instead is associated with a lower frequency of cleaved caspase 3 positive cells suggesting that constitutive activation of Stat3 confers enhanced survival to this TEC subset. Furthermore, the MHC II^lo^CD80^lo^ Aire^-^ TECs in K5.Stat3C thymi express low levels of CD80 and are involucrin negative, indicating that these cells do not represent a post-Aire differentiation stage and supporting the notion that they are immature mTEC precursors. Although MHC II^lo^CD80^lo^ Aire^-^ mTEC cellularity was increased, there was not a significant change in the number of mature MHC II^hi^CD80^hi^ Aire^+^ mTECs despite the fact that the transgene is expressed and increased levels of phosphorylated Stat3 are present in mature MHC II^hi^ mTECs. Although a higher turnover rate may prevent accumulation of transgenic MHC II^hi^CD80^hi^ Aire^+^ mTECs, this explanation seems unlikely given that there was not a higher frequency of proliferating or caspase positive cells in the MHC II^hi^CD80^hi^ subset. It is possible that a block in progenitor differentiation restricts the number of mature mTECs. However, the similar number of MHC II^hi^CD80^hi^ mTECs in K5.Stat3C transgenic and NTg control thymi demonstrates that at least some of the MHC II^lo^CD80^lo^ mTEC subset in K5.Stat3C thymi can differentiate to the MHC II^hi^CD80^hi^ stage. We speculate that limited availability of microenvironmental niches in K5.Stat3C thymi restricts the number of mTEC progenitors that can complete maturation to the MHC II^hi^CD80^hi^ mTEC stage. Although further investigation is required to clarify the role of Stat3 signaling in mTEC differentiation, the presence of MHCII^hi^ Aire positive mTECs in the expanded medullary regions of RAG-2^-/-^;K5.Stat3C mice is consistent with the possibility that Stat3 activation may play a role in mTEC differentiation.

Collectively, the data from this investigation and the accompanying report (Satoh *et al*.) demonstrate that Stat3 activation promotes mTEC survival and is essential for optimal medullary region development and homeostasis. Thus, in addition to the well-characterized requirement for canonical and noncanonical NFκB mediated signaling pathways, Stat3-mediated signaling contributes to optimal development and maintenance of the thymic medullary compartment.

## Materials and Methods

### Ethics statement

MD Anderson Cancer Center IACUC approved the research performed with mice. The approval number is 00001097-RN00. MD Anderson is accredited by the Association for Assessment and Accreditation of Laboratory Animal Care (AAALC). The AAALC number is 000183.

After termination of the experiments, animals were euthanized in a CO_2_ chamber.

### Mice

K5.Stat3C transgenic mice and K5.Cre;Stat3^fl/fl^ conditional knockout mice were generated and characterized as described previously [50, 76, 77]. Stat3^fl/fl^ mice and Stat3^fl/wt^ mice were littermate controls. Mice were maintained at the MD Anderson Cancer Center Department of Molecular Carcinogenesis in accordance with the guidelines set forth by the Association for the Accreditation of Laboratory Animal Care.

### Morphometric analysis

Sections from 6–10 week old thymi were obtained from the center of the thymus over a 200 um distance, discarding 50 um between each depth. Ten sections were analyzed from each individual with 2 sections taken at each depth to provide a reliable representation of each thymus. Sections were stained with hematoxylin-eosin and scanned using Aperio Scanscope hardware. The digital copies of whole sections were saved and processed with Image Scope software from Aperio. The medullary and cortical areas were normalized against the area of the entire thymic section. A medulla/cortex classifier algorithm was employed to calculate relative medullary and cortical areas, expressed as the medulla/cortex ratio.

### Flow cytometry

Single cell suspensions of TECs were obtained collagenase (Collagenase D type IV, Worthington) and dispase (Roche) enzymatic dissociation as previously described [78] and thymocytes were obtained by pressing the thymus through a 70μm strainer (Fisher). Cells were stained with fluorochrome-conjugated antibodies in FACS buffer (PBS pH 7.2, 0.005M EDTA, 2% FBS) for 20 minutes on ice and washed. Propidium iodide (Invitrogen) was added (0.5 μg/ml) to each sample prior to analysis to exclude dead cells. Anti-CD326 (clone G8.8), anti-I-A/I-E (clone M5/114.15.2), anti-CD25 (clone PC61) and anti-CD117 (clone 2 B8) were purchased from Biolegend. Anti-CD44 (clone IM7), anti-CD8α (clone 53–6.7), anti-CD45 (clone 30-F11) and anti-Bcl-2 (clone 3F11) were purchased from eBioscience. Biotinylated anti-Ly51, anti-pStat3 (clone 4/p-Stat3 pY705) and anti-total Stat3 (clone M59-50) were purchased from BD Biosciences. Anti-CD4 (clone RM4-5) and Streptavidin Qdot 655 was purchased from Invitrogen. To exclude erythrocytes, granulocytes, dendritic cells, macrophages and NK cells from the thymocyte analyses, the following antibodies were purchased from eBioscience: TER-119, CD11c (cloneN418), CD11b (clone M1/70), NK-1.1 (clone PK136) and Ly-6G (clone RB6-8C5). Flow cytometry was performed on a Becton Dickenson FACS Aria II and data were analyzed using FlowJo software (Tree Star).

### Immunohistochemistry

Serial sections (5 μm) from OCT-embedded frozen tissue were air dried and fixed briefly in cold acetone. Thymic sections were incubated with rabbit polyclonal antibodies to K5, K14 or involucrin (Covance) or with anti-Aire1 (Santa Cruz Biotechnology, Inc). Primary monoclonal antibodies included rat anti-K8 (Troma-1 Developmental Studies Hybridoma Bank), biotinylated anti-CD11c (BD Biosciences), and anti-CD326 (clone G8.8 from Biolegend). UEA-1 lectin (Vector Laboratories) was obtained as a biotinylated reagent. Secondary reagents included donkey anti-rabbit Ig, donkey anti-rat Ig, goat anti-rabbit (Jackson ImmunoResearch) and FITC-conjugated streptavidin (Invitrogen). Staining for CD11c was enhanced using tyramide amplification. Microscopic analysis and image capture was performed using an Olympus AX700 microscope.

### Proliferation and apoptosis assays

Mice were injected by the i.p. route with 1mg BrdU (Sigma-Aldrich) in PBS and continuous labeling was maintained by providing 0.8 mg/ml BrdU in 1% sucrose solution for 72 hr. TECs were incubated with antibodies to surface antigens and fixed overnight at 4°C using Fix/Perm buffer (BD Biosciences). The cells were permeabilized with 1% paraformaldehyde/0.5% Tween-20/PBS for 30 min at room temperature, followed with re-fixing using Fix/Perm buffer for 15 min at 4°C. Following DNAse treatment (30 μg/sample) for 1h at 37°C, the cells were incubated with FITC conjugated anti-BrdU antibody for 30 min at 4°C. To analyze apoptosis, TECs were fixed and permeabilized as for BrdU analysis above, incubated with rabbit anti-cleaved caspase 3 (Cell Signaling) or an isotype control and developed with anti-rabbit Alexa Fluor 488 (Molecular Probes).

### Western blot analysis

Protein lysates were made from FACS sorted CD45^-^ stromal cells and CD45^+^ hematopoietic cells using RIPA lysis buffer. The supernatant was separated by electrophoresis on 8–12% SDS-PAGE gels, electrophoretically transferred onto PVDF membranes and blocked with 5% non-fat dry milk in PBS with 0.1% Tween 20 for 1 hr at RT. Blots were incubated for 2 hr at RT with specific primary antibodies for Flag, phospho-Stat3, Stat3 and tubulin (Cell Signaling),were washed in PBS with 0.1% Tween 20 and incubated with HRP-conjugated secondary antibodies against rabbit or mouse (Bio-Rad). Blots were washed with PBS with 0.1% Tween 20 and detected with ECL Western Blotting kit (Pierce).

### Quantitative real time PCR

Total RNA was purified from FACS sorted mTECs, cTECs and CD45^+^ hematopoietic cells and was purified using TRIzol (Life Technologies). cDNA was synthesized using a high capacity RT kit from Applied Biosystems following manufacturer’s instructions. qPCR was performed with iTAQ SYBR Green Supermix (Bio-rad) or TaqMan master mix (Applied Biosystems). Reactions were run on ABI Prism 7900 HT and gene expression was normalized to endogenous α-tubulin (Mm00846967_g1, Life Technologies). The mouse primer sequences used given in S1 Table.

## Supporting Information

S1 Fig Vasculature in RAG2^-/-^ and RAG2^-/-^;K5.Stat3C thymi. IHC stains of RAG2^-/-^ and RAG2^-/-^;K5.Stat3C frozen thymus sections show vasculature detected by VE-cadherin positive cells and medullary regions detected by K14 positive cells. White arrows show K14 bounded cyst-like structures.
(TIFF)

S2 Fig K5.Stat3C transgene expression and pStat3 levels in TEC subsets from NTg and K5.Stat3C thymi. **(A)** Quantitative RT-PCR analysis of Flag expression in FACS sorted K5.Stat3C and NTg TEC subsets. Representative data from two independent experiments with duplicate or triplicate samples in each experiment. **(B)** FACS histograms of phosphorylated Stat3 levels in K5.Stat3C and NTg TEC subsets. Data are representative of 3 experiments.
(TIFF)

S3 Fig Increased frequency of MHCII^lo^CD40^lo^ mTECs in K5.Stat3C thymi. Representative FACS plots show K5.Stat3C mTECs contain an increased percentage of MHCII^lo^CD40^lo^ cells compared to NTg controls.
(TIFF)

S4 Fig Increased numbers of immature and mature mTECs in RAG2^-/-^;K5.Stat3C transgenic thymi. **(A)** Bar graph (mean ± SD) shows cellularity of total TECs, cTECs, immature and mature mTECs in RAG2^-/-^ compared to RAG2^-/-^;K5.Stat3C thymi (n = 3 each). **(B)** Representative IHC stains of K14+ and Aire+ cells in small medullary foci of RAG2^-/-^ thymi and expanded medullary regions of RAG2^-/-^;K5.Stat3C thymi.
(TIFF)

S5 Fig TRA expression by mTECs and comparative histological analysis of organs from K5.Stat3C and control mice. **(A-C)** Quantitative RT-PCR analysis of MHCII^hi^ mTECs from K5.Stat3C and control thymi shows comparable expression of **(A)** *Aire* **(B)** Aire-dependent TRAs, and **(C)** Aire-independent TRAs normalized to α-tubulin mRNA. The NTg control was set at 1. Bar graphs show mean ± SEM of 3 independent experiments with duplicate or triplicate samples in each experiment. **(D)** H&E stained tissue sections of kidney, lacrimal gland, liver and pancreas in K5.Stat3C and controls.
(TIFF)

S6 Fig Absence of autoantibodies in K5.Stat3C serum. Sera from K5.Stat3C and NTg mice were tested for the presence of autoantibodies (red) by incubating on tissue sections from *Rag2*^*-/-*^ mice. Nuclei were detected with DAPI (blue). Serum from *CCR7*^*-/-*^ mice served as a positive control.
(TIFF)

S7 Fig PCR analysis of K5.Cre mediated *Stat3* deletion. **(A)** PCR analysis was performed on tail DNA from mice of the indicated genotypes or from *K5*.*Cre;Stat3*^*fl/fl*^ total TECs. Arrows indicate bands corresponding to wildtype, floxed or deleted *Stat3* alleles. **(B)** PCR analysis of the 650bp ΔStat3 band in FACS sorted cTECs and mTEC subsets from control and Stat3 CKO thymi. **(C)** FACS histograms of Stat3 levels in Stat3 CKO and control TEC subsets. Data are representative of 2 experiments.
(TIFF)

S8 Fig Thymocyte subsets in Stat3 CKO mice. **(A)** Bar graphs showing the percentage of major thymocyte subsets defined by CD4 and CD8 expression or DN subsets defined by CD44 and CD25 expression (n = 4 for all). **(B)** Bar graphs showing the number (mean ± SD) of major thymocyte subsets defined by CD4 and CD8 expression or of DN subsets defined by CD44 and CD25 expression (n = 4).
(TIFF)

S9 Fig Ratio of medulla to cortex in Stat3 CKO and control mice. Medulla/cortex ratio in Stat3 CKO (n = 3) and control (n = 3) thymi as determined by morphometric analysis of Aperio scanned H&E stained sections.
(TIFF)

S10 Fig TRA expression by mTECs and comparative histological analysis of organs from Stat3 CKO and control mice. **(A-C)** Quantitative RT-PCR analysis of sorted MHCII^hi^ mTECs from Stat3 CKO and control thymi shows comparable expression of **(A)** Aire-dependent TRAs and **(B)** Aire-independent TRAs normalized to α-tubulin mRNA. The wildtype control was set at 1. Bar graphs show mean ± SEM of three independent experiments with duplicate or triplicate samples in each experiment. **(C)** H&E stained tissue sections of kidney, lacrimal gland, liver and pancreas in Stat3 CKO and control mice. Scales bars equal 500um in kidney, and 200um in other images, as indicated.
(TIFF)

S11 Fig Absence of autoantibodies in Stat3 CKO serum. Sera from Stat3 CKO and control mice were tested for the presence of autoantibodies (red) by incubating on tissue sections from *Rag2*^*-/-*^ mice. Nuclei were detected with DAPI (blue). Serum from *CCR7*^*-/-*^ mice served as a positive control.
(TIFF)

S1 Table qRT-PCR primer sequences. (PDF)
