# Supplementary figures and images for: Stat3 Signaling Promotes Survival And Maintenance Of Medullary Thymic Epithelial Cells
Source: PLoS Genet. 2016 Jan 20;12(1):e1005777. doi: 10.1371/journal.pgen.1005777 (PMC4720390; doi:10.1371/journal.pgen.1005777)

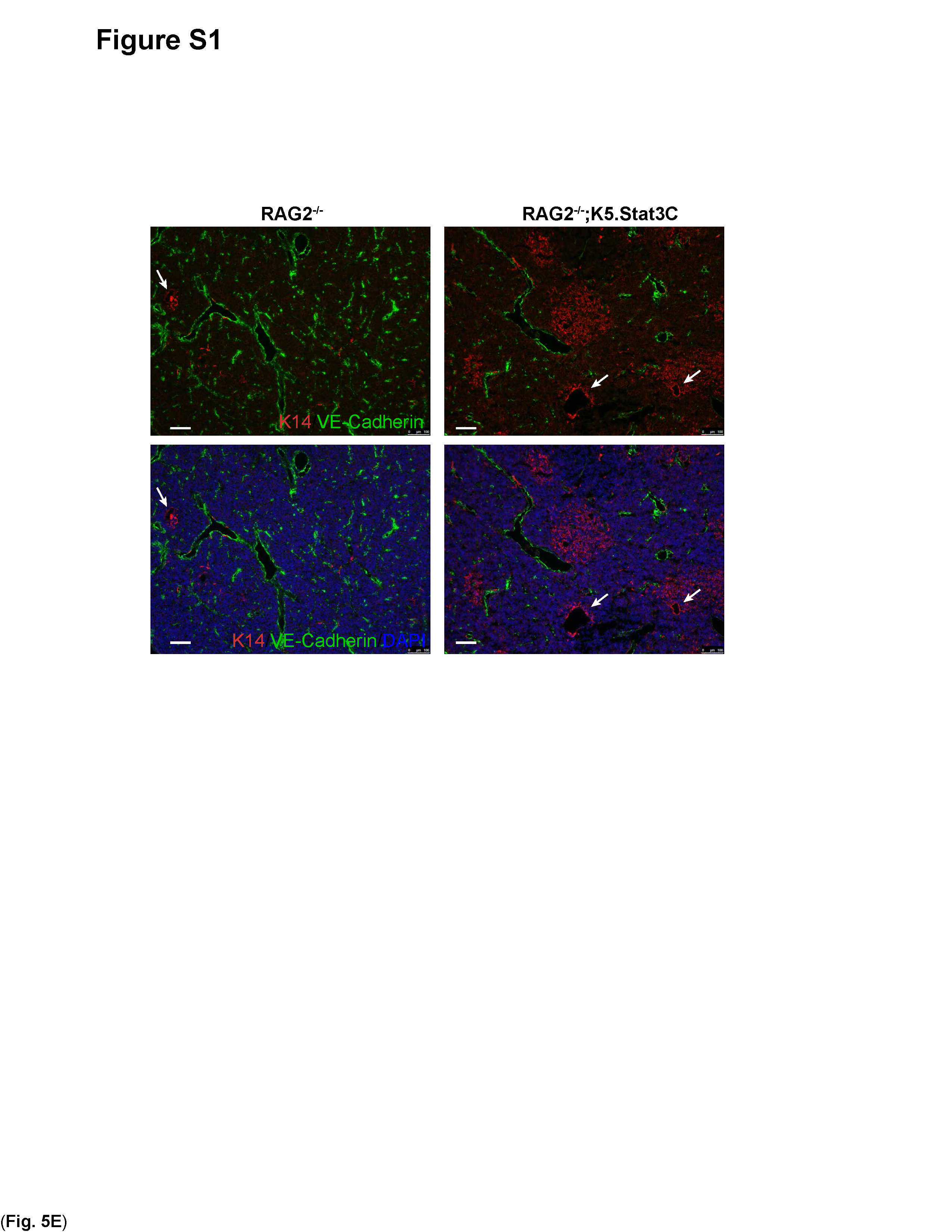

Supplement: S1 Fig — IHC stains of RAG2-/- and RAG2-/-;K5.Stat3C frozen thymus sections show vasculature detected by VE-cadherin positive cells and medullary regions detected by K14 positive cells. White arrows show K14 bounded cyst-like structures. (TIFF) [file pgen.1005777.s001.tiff]

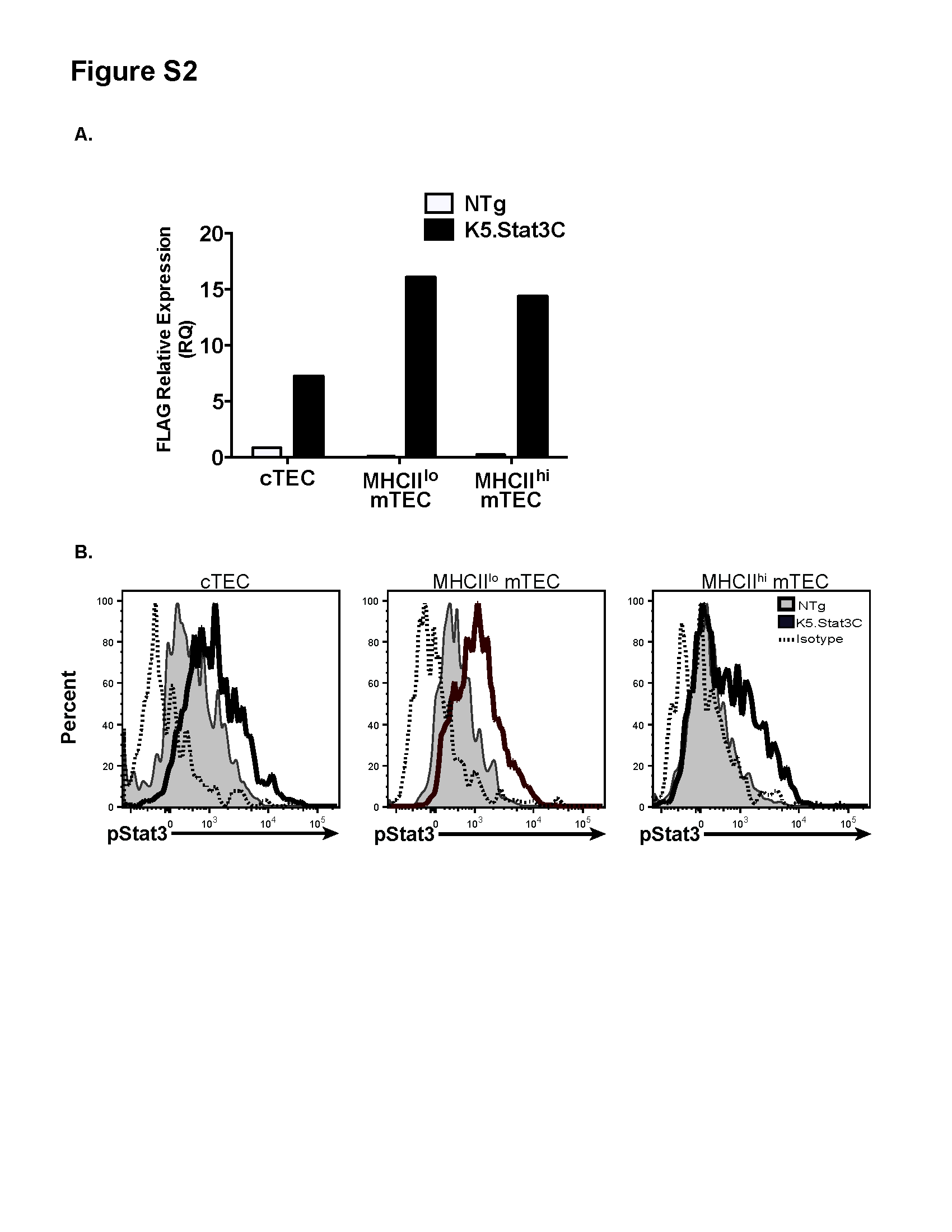

Supplement: S2 Fig — (A) Quantitative RT-PCR analysis of Flag expression in FACS sorted K5.Stat3C and NTg TEC subsets. Representative data from two independent experiments with duplicate or triplicate samples in each experiment. (B) FACS histograms of phosphorylated Stat3 levels in K5.Stat3C and NTg TEC subsets. Data are representative of 3 experiments. (TIFF) [file pgen.1005777.s002.tiff]

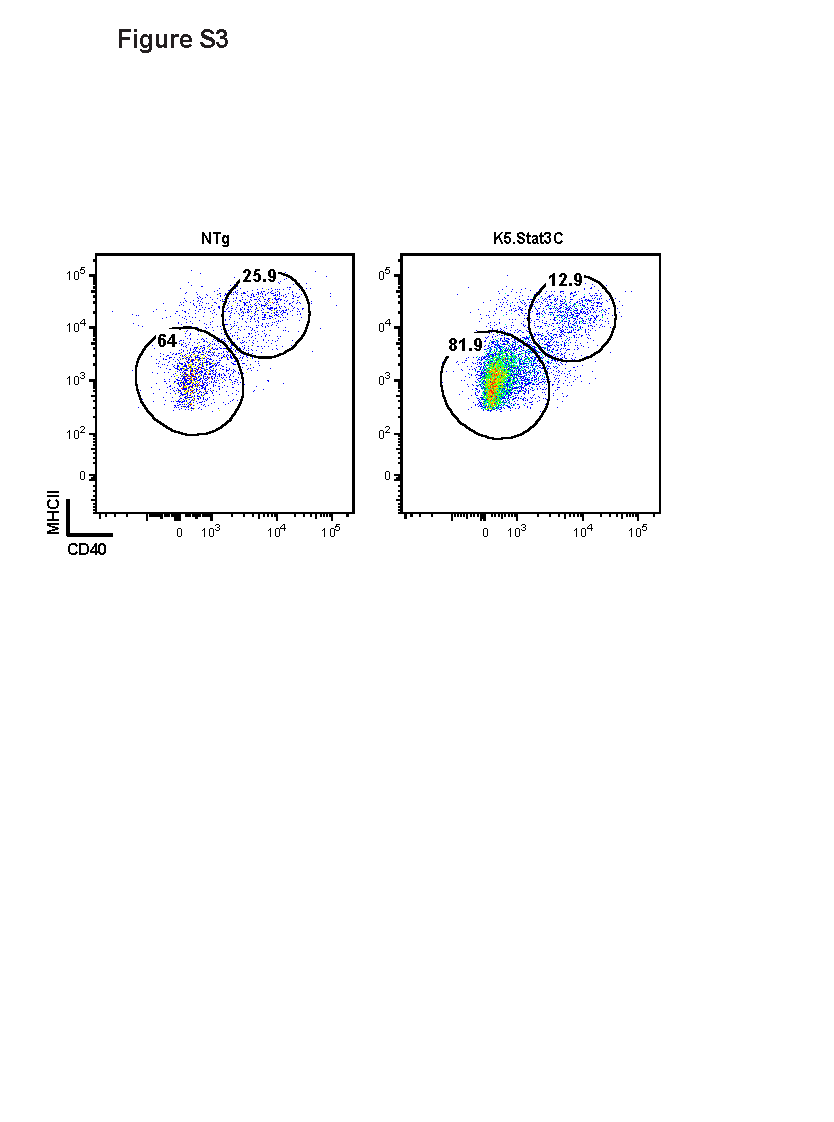

Supplement: S3 Fig — Representative FACS plots show K5.Stat3C mTECs contain an increased percentage of MHCIIloCD40lo cells compared to NTg controls. (TIFF) [file pgen.1005777.s003.tiff]

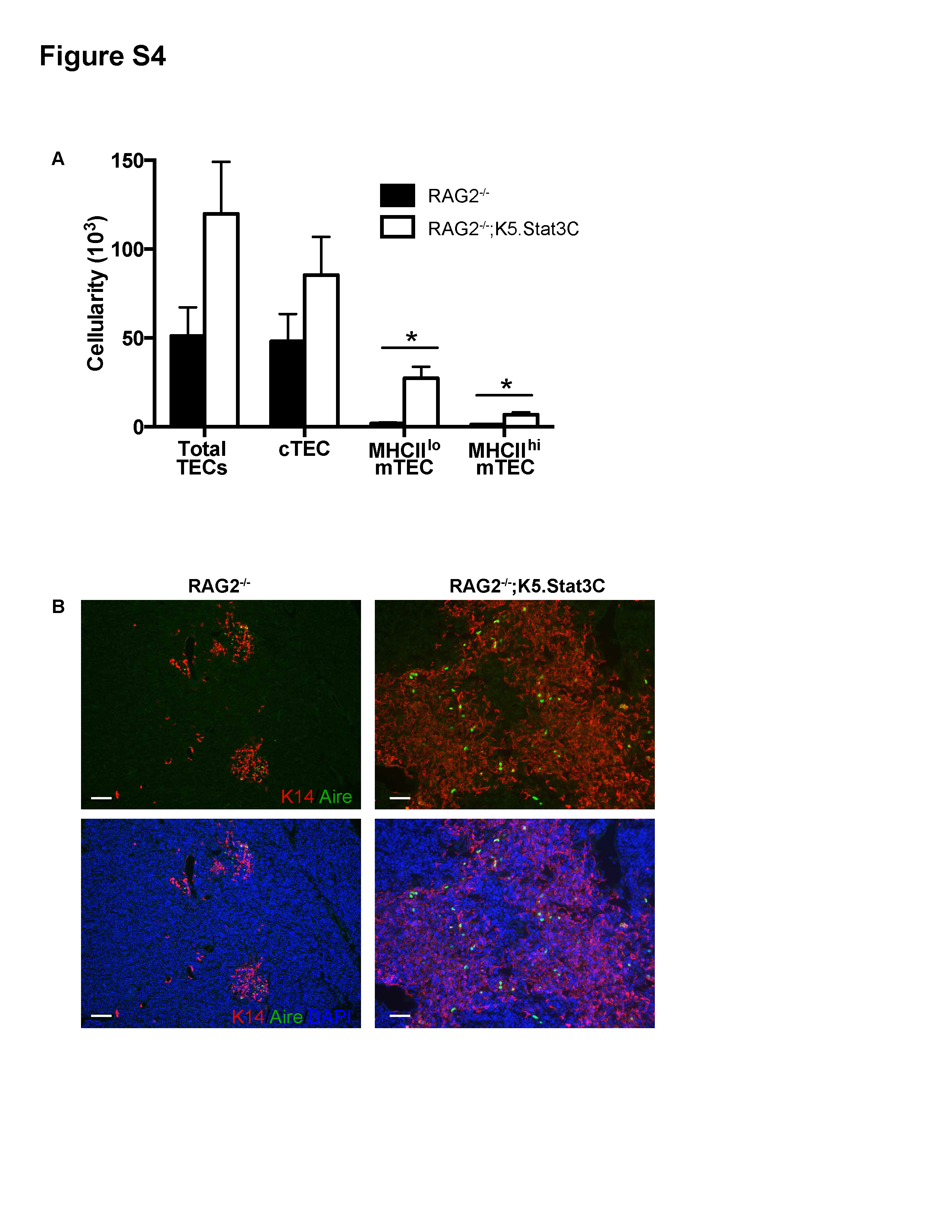

Supplement: S4 Fig — (A) Bar graph (mean ± SD) shows cellularity of total TECs, cTECs, immature and mature mTECs in RAG2-/- compared to RAG2-/-;K5.Stat3C thymi (n = 3 each). (B) Representative IHC stains of K14+ and Aire+ cells in small medullary foci of RAG2-/- thymi and expanded medullary regions of RAG2-/-;K5.Stat3C thymi. (TIFF) [file pgen.1005777.s004.tiff]

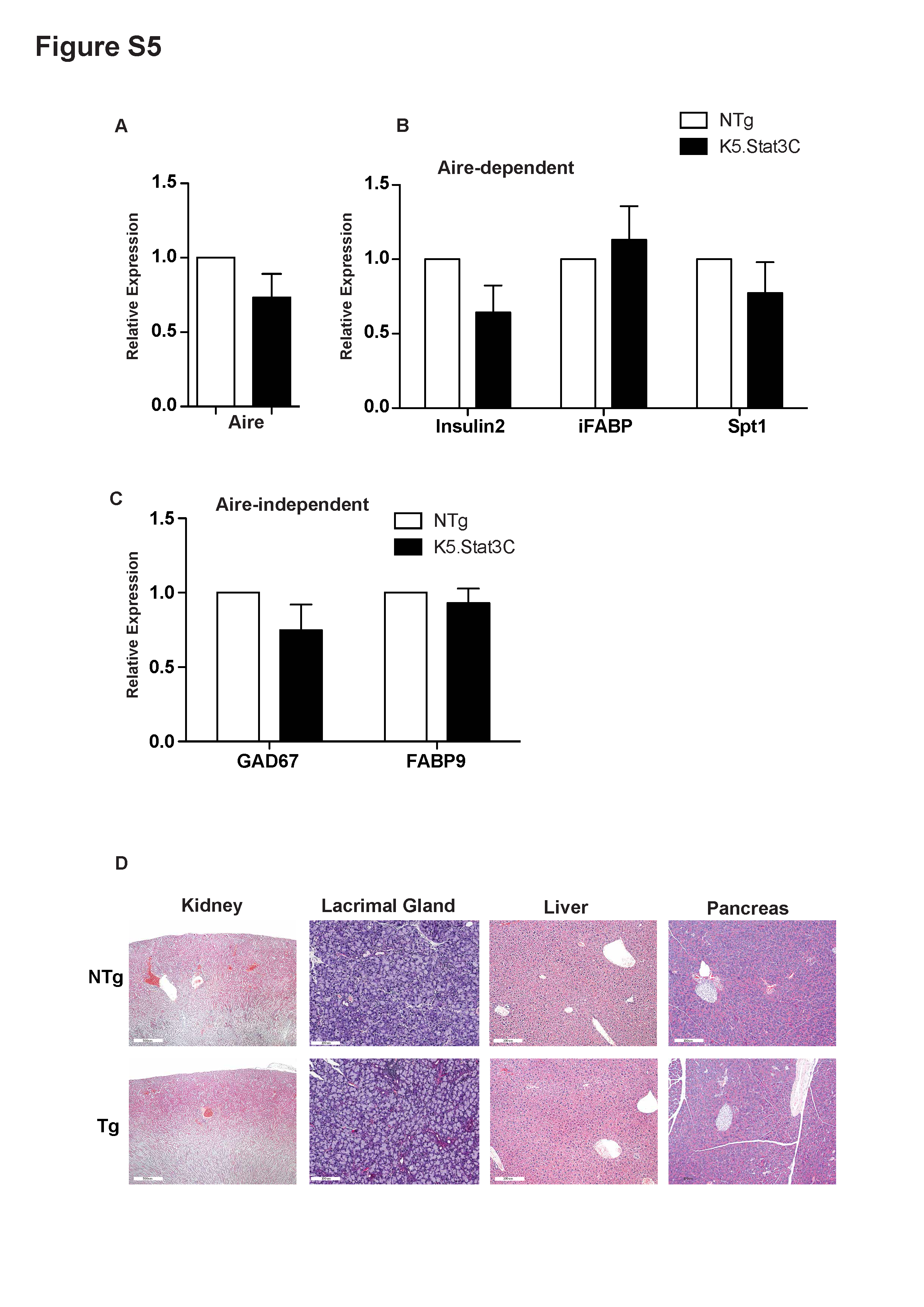

Supplement: S5 Fig — (A-C) Quantitative RT-PCR analysis of MHCIIhi mTECs from K5.Stat3C and control thymi shows comparable expression of (A) Aire (B) Aire-dependent TRAs, and (C) Aire-independent TRAs normalized to α-tubulin mRNA. The NTg control was set at 1. Bar graphs show mean ± SEM of 3 independent experiments with duplicate or triplicate samples in each experiment. (D) H&E stained tissue sections of kidney, lacrimal gland, liver and pancreas in K5.Stat3C and controls. (TIFF) [file pgen.1005777.s005.tiff]

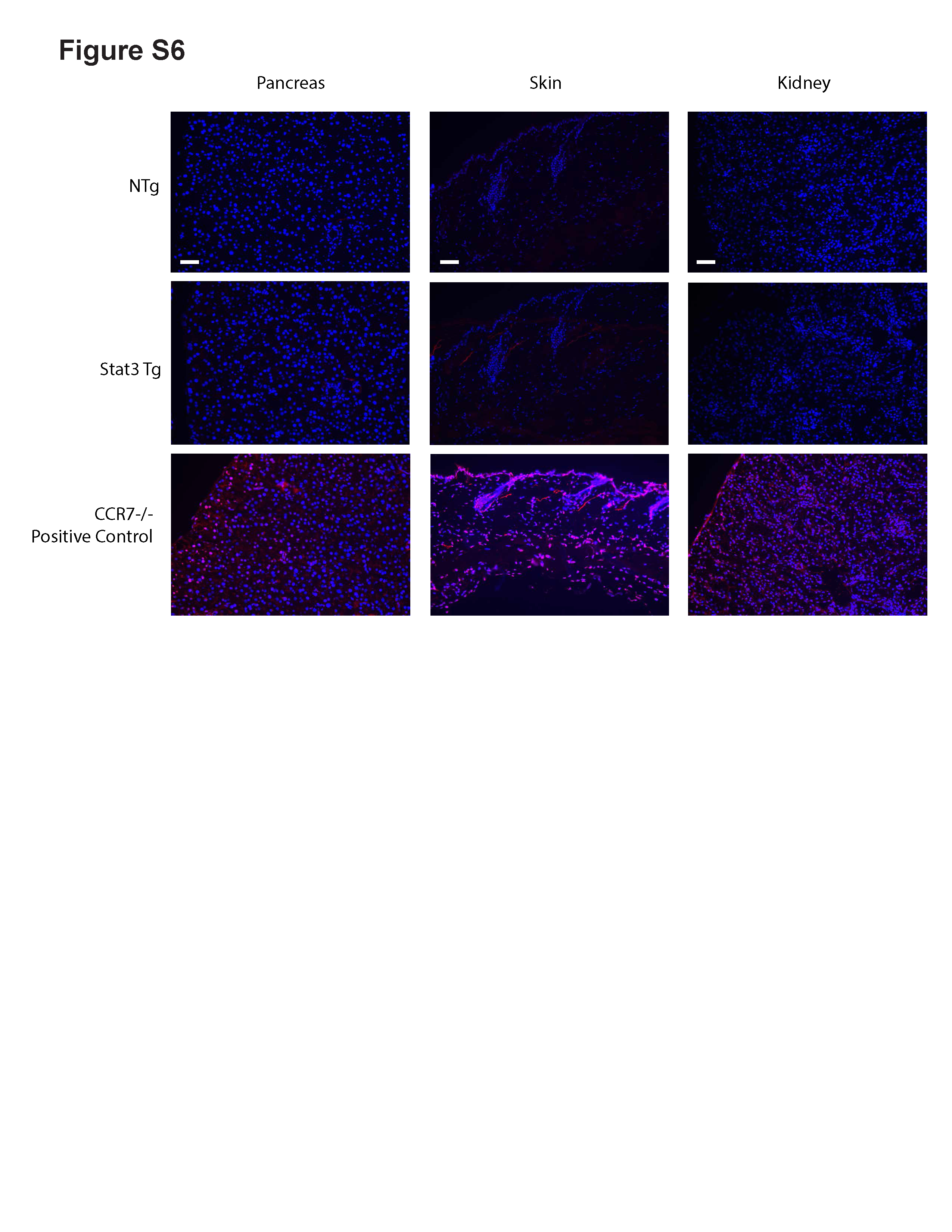

Supplement: S6 Fig — Sera from K5.Stat3C and NTg mice were tested for the presence of autoantibodies (red) by incubating on tissue sections from Rag2-/- mice. Nuclei were detected with DAPI (blue). Serum from CCR7-/- mice served as a positive control. (TIFF) [file pgen.1005777.s006.tiff]

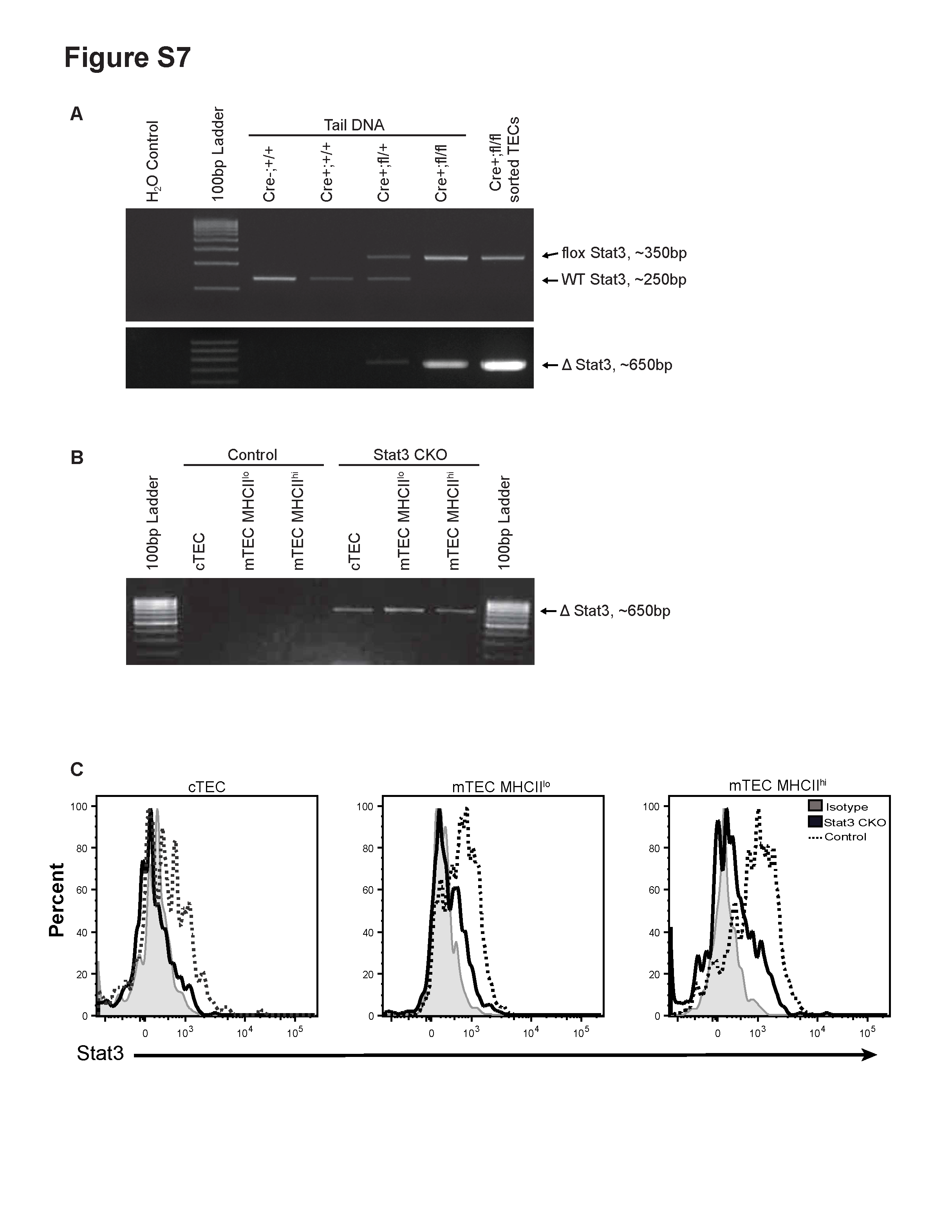

Supplement: S7 Fig — (A) PCR analysis was performed on tail DNA from mice of the indicated genotypes or from K5.Cre;Stat3fl/fl total TECs. Arrows indicate bands corresponding to wildtype, floxed or deleted Stat3 alleles. (B) PCR analysis of the 650bp ΔStat3 band in FACS sorted cTECs and mTEC subsets from control and Stat3 CKO thymi. (C) FACS histograms of Stat3 levels in Stat3 CKO and control TEC subsets. Data are representative of 2 experiments. (TIFF) [file pgen.1005777.s007.tiff]

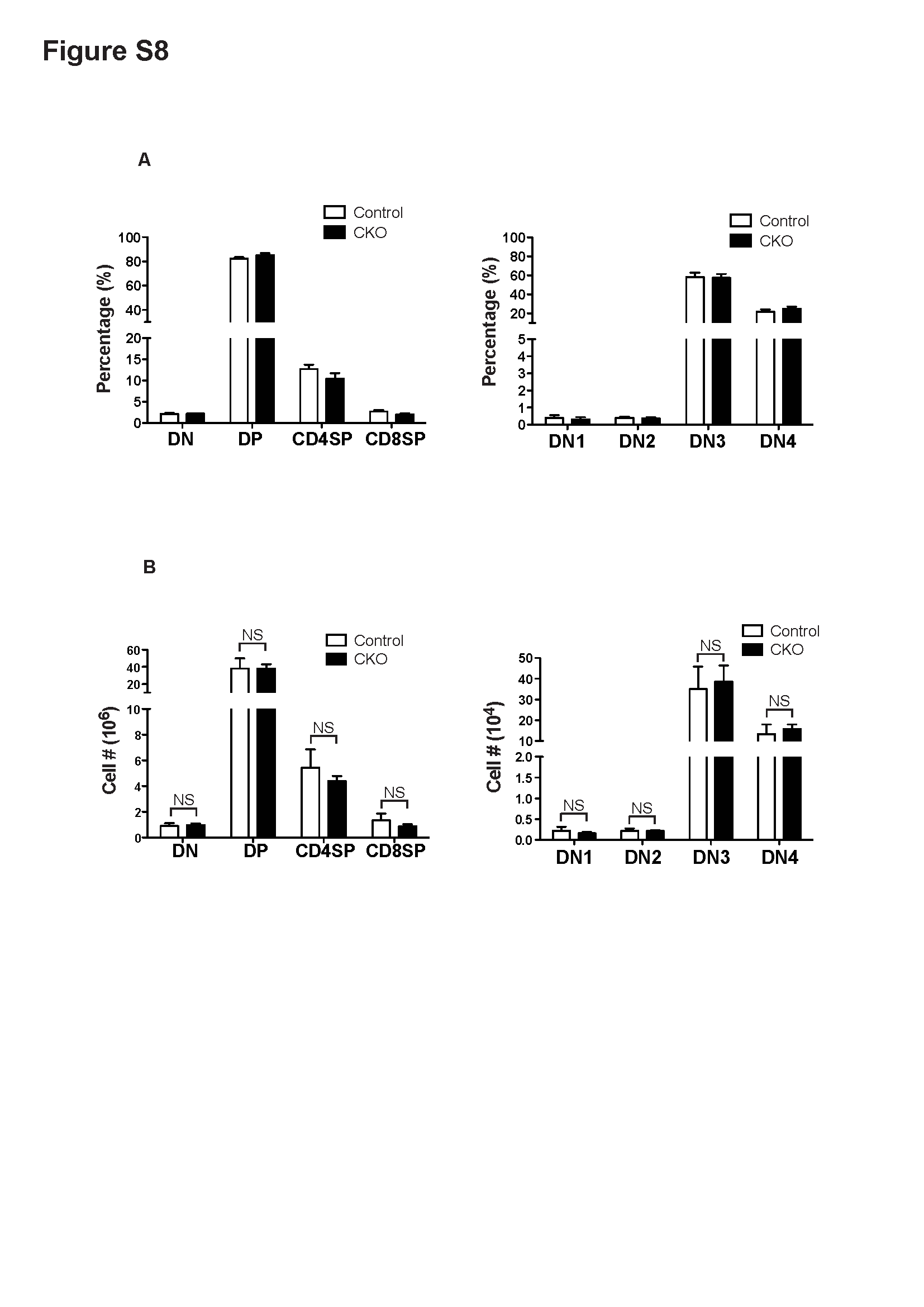

Supplement: S8 Fig — (A) Bar graphs showing the percentage of major thymocyte subsets defined by CD4 and CD8 expression or DN subsets defined by CD44 and CD25 expression (n = 4 for all). (B) Bar graphs showing the number (mean ± SD) of major thymocyte subsets defined by CD4 and CD8 expression or of DN subsets defined by CD44 and CD25 expression (n = 4). (TIFF) [file pgen.1005777.s008.tiff]

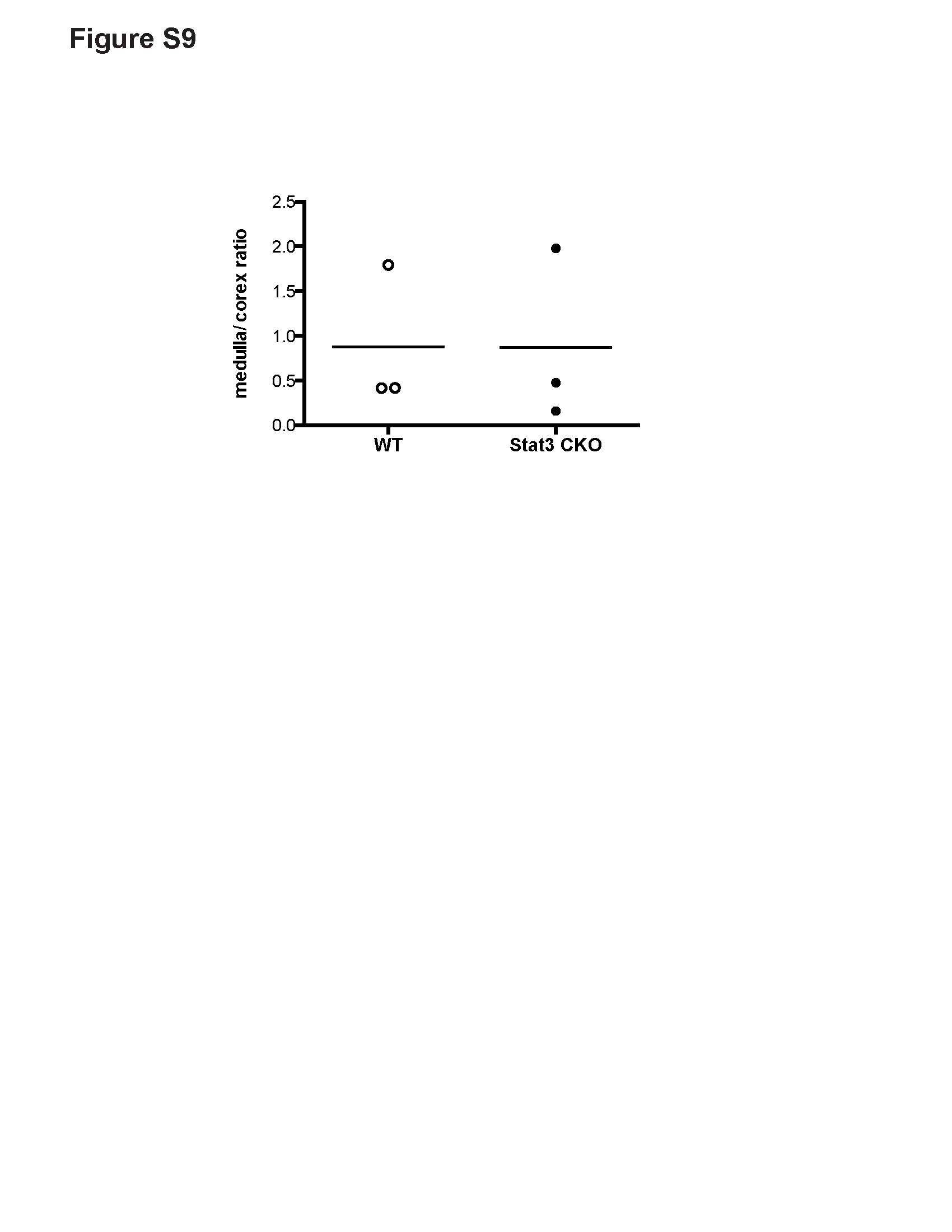

Supplement: S9 Fig — Medulla/cortex ratio in Stat3 CKO (n = 3) and control (n = 3) thymi as determined by morphometric analysis of Aperio scanned H&E stained sections. (TIFF) [file pgen.1005777.s009.tiff]

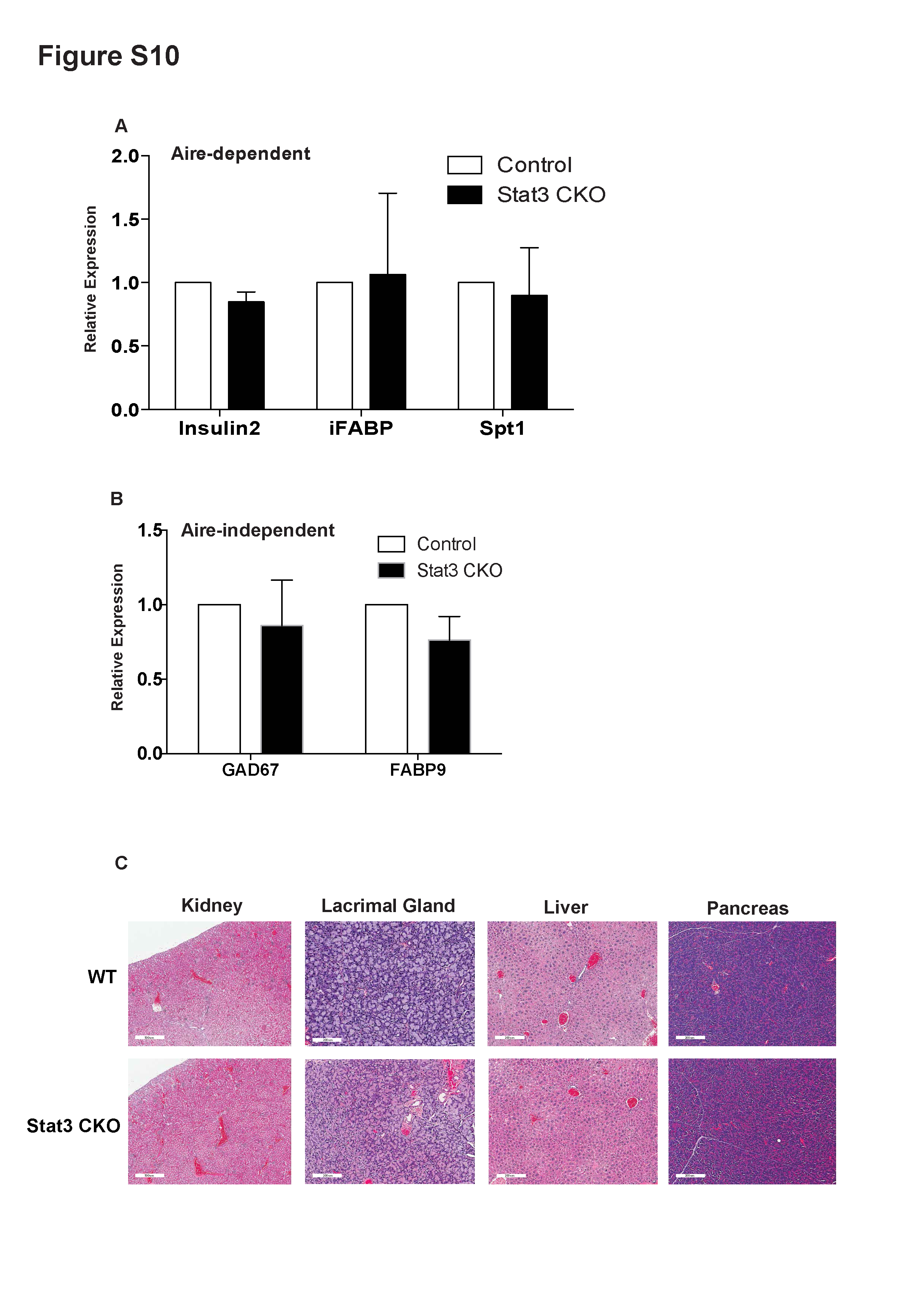

Supplement: S10 Fig — (A-C) Quantitative RT-PCR analysis of sorted MHCIIhi mTECs from Stat3 CKO and control thymi shows comparable expression of (A) Aire-dependent TRAs and (B) Aire-independent TRAs normalized to α-tubulin mRNA. The wildtype control was set at 1. Bar graphs show mean ± SEM of three independent experiments with duplicate or triplicate samples in each experiment. (C) H&E stained tissue sections of kidney, lacrimal gland, liver and pancreas in Stat3 CKO and control mice. Scales bars equal 500um in kidney, and 200um in other images, as indicated. (TIFF) [file pgen.1005777.s010.tiff]

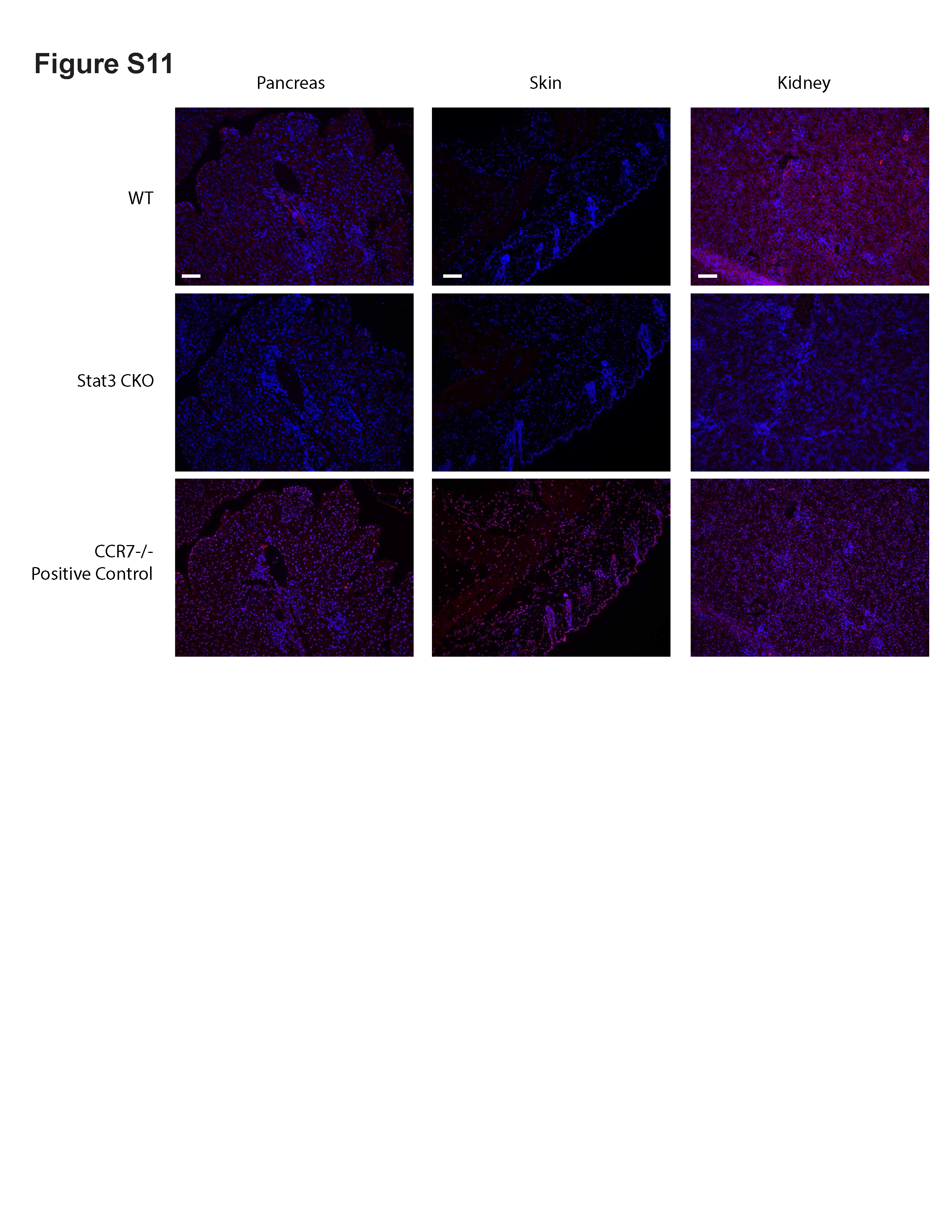

Supplement: S11 Fig — Sera from Stat3 CKO and control mice were tested for the presence of autoantibodies (red) by incubating on tissue sections from Rag2-/- mice. Nuclei were detected with DAPI (blue). Serum from CCR7-/- mice served as a positive control. (TIFF) [file pgen.1005777.s011.tiff]
