# Supplementary material for: Stat3 Signaling Promotes Survival And Maintenance Of Medullary Thymic Epithelial Cells
Source: PLoS Genet. 2016 Jan 20;12(1):e1005777. doi: 10.1371/journal.pgen.1005777 (PMC4720390; doi:10.1371/journal.pgen.1005777)
Supplement: S1 Table — (PDF) [file pgen.1005777.s012.pdf]

Table S1. qRT-PCR primer sequences

Insulin 2	5'-CCGCTACAATCAAAAACCATCAGCAAG-3’
	5'ATCTACAATGCCACGCTTCTGCTGG3’
Spt1	5'-TCCTTGTGTTGCTTGGTGTTTCCAC-3’
	5'-GCATTGGACTCGTCTTCCGTTTCAG-3’
FABP9	5'-TGGTTTTCGGTTGTGAATGCCTG-3’
	5'-TCTTGCTGGTGTCTACCCTTCTGCTAC-3’
GAD67	5'-TGACACCGGGGACAAGGCGAT-3’
	5'-TCTTGGGAGCCACCCTGTGTAGC-3’
iFABP	5'-GAGATCATGGCATTCGACGGCACG-3’
	5'-TGGTCCAGGCCCCAGTGAGCTC-3’
Aire,	5'-TCTGCTAGTCACGACCCTGTTC-3’
	5'-GCAGGATGCCGTCAAATGAGTG-3’
Bcl2	5'-TGTGTGTGGAGAGCGTCAACAG-3’
	5'-CGGTTCAGGTACTCAGTCATCCAC-3’.
